# Design and Validation of a Predictive Model for Hepatocellular Carcinoma Based on Genes With Differential Expression Driven by DNA Methylation

**DOI:** 10.1155/ijog/2729004

**Published:** 2026-01-27

**Authors:** Geyang Hu, Liang Zhou, Jie Zhang

**Affiliations:** ^1^ Bengbu Medical College Graduate Department, Bengbu, Anhui, China; ^2^ Department of Hepatological, Affiliated Hospital of Jiaxing University, Jiaxing, Zhejiang, China; ^3^ Department of Cardio-Thoracic Surgery, Affiliated Hospital of Jiaxing University, Jiaxing, Zhejiang, China

**Keywords:** DNA methylation, drug resistance, epigenetics, hepatocellular carcinoma, prognosis

## Abstract

**Background:**

Hepatocellular carcinoma (HCC) ranks among the world′s most lethal cancers, with the majority of cases diagnosed at advanced stages. Accurate prognostic assessment is therefore essential for HCC management. This study utilized DNA methylation (MDGs) and RNA‐sequencing data to develop and validate a predictive model for HCC.

**Methods:**

MDG profiles, RNA‐seq data, and related clinical information were analyzed. Based on the Cancer Genome Atlas (TCGA) dataset, a prognostic signature was developed via univariable and multivariable Cox regression analyses in combination with LASSO regression. Subsequently, a nomogram model was constructed and calibrated using calibration curves. The predictive accuracy of the selected genes was tested through in vitro cellular experiments. In addition, the GDSC dataset was utilized to examine the association between the prognostic signature and drug resistance.

**Results:**

Three genes (GLS, TEAD4, and CLGN) were identified and incorporated into the prognostic signature. Low‐risk patients exhibited notably improved overall survival (OS) in comparison to high‐risk patients. A nomogram model was developed based on clinical variables associated with OS, and its predictive accuracy for OS in individuals with HCC was evaluated via calibration curves. In vitro experiments revealed that the proliferative capacity of cells was notably reduced in the knockout group. The GDSC database was utilized to examine the association between the identified prognostic features and drug resistance.

**Conclusion:**

Predictive risk scores were developed based on three candidate MDGs, and a nomogram model was built by integrating clinical variables with these scores. This model can provide personalized prognosis prediction and assess drug resistance among individuals with HCC.

## 1. Introduction

Hepatocellular carcinoma (HCC) is one of the most aggressive gastrointestinal cancers globally, ranking as the fourth leading cause of cancer deaths and the sixth most commonly diagnosed malignancy worldwide [1–3]. Viral hepatitis, particularly Hepatitis B virus (HBV), is one of the primary causes of HCC [4]. Notably, China accounts for nearly half of global liver cancer (LC) cases. Thus, it is necessary to develop effective therapeutic strategies for LC [5]. Advances in early detection, interventional therapy, and innovations in surgical and therapeutic techniques have improved clinical outcomes. For example, targeted and immunotherapy have transformed the treatment landscape for advanced HCC, marking a new phase in systemic therapy [6, 7]. Nevertheless, many individuals with HCC are diagnosed at an advanced stage, leading to poor overall survival (OS) [8, 9]. Elucidating the molecular mechanisms and regulatory pathways in HCC is critical for identifying novel biomarkers for early screening, prognosis, and recurrence monitoring [10].

Epigenetics represents heritable modifications that alter gene function without altering the DNA sequence, primarily by modulating chromatin structure and dynamics [11]. These modifications, including changes in noncoding DNA, DNA methylation (MDGs), and histone acetylation, are widely recognized as heritable alterations that influence gene expression [12]. MDGs serve as a key epigenetic modification that preserves genome stability and modulates gene transcription. It typically occurs through the addition of methyl groups (–CH3) to the fifth carbon of cytosine in CpG dinucleotides, resulting in the formation of 5‐methylcytosine [13]. A growing body of research has demonstrated that MDGs are a key contributor to hepatocarcinogenesis. A study by Wang et al. found positive relationships between the methylation of the retinoic acid–inducible gene I (RIG‐I) and steatosis and hepatocarcinogenesis triggered by NASH. Moreover, the demethylation of RIG‐I by JMJD4 suppresses necroinflammation and tumor development induced by NASH [14]. A study by Zhang et al. discovered that MDGs may elevate the risk of HCC related to the Hepatitis C virus [15], suggesting their role as a biomarker. Aberrant MDGs lead to dysregulated expression of numerous tumor‐related genes, thereby contributing to tumor progression. In particular, hypermethylation of tumor suppressor genes and hypomethylation of oncogenes are key drivers of cancer development, including HCC [16–18]. Existing research did not systematically combine MDG microarray data and RNA‐sequencing (RNA‐seq) data to identify prognostically relevant molecular features in HCC. Identifying MDGs and key biomarkers is essential for predicting HCC prognosis. In this study, differentially expressed genes (DEGs) and MDGs in HCC were obtained by incorporating transcriptomic and MDG data. Subsequently, a prognostic signature incorporating three selected key genes was established, and the GEO dataset was utilized to validate its performance. A nomogram incorporating the risk score derived from MDGs and clinicopathologic risk factors was established to assess OS among individuals with HCC. These findings may help enhance prognostic assessment in HCC.

Methylation is marked by the addition of a –CH3 to DNA, primarily at cytosine residues within CpG dinucleotides. This fundamental epigenetic modification can regulate gene expression and various cellular processes. HCC, the most common type of LC, exhibits abnormal MDG patterns that may contribute to the development of drug resistance. Drug resistance is defined as reduced or absent responsiveness of cancer cells to anticancer drugs. This resistance may arise from diverse mechanisms, including altered drug metabolism, enhanced drug efflux, DNA repair processes, and aberrant cellular signaling pathways. MDGs influence drug response in tumor cells by regulating gene expression, thereby contributing to drug resistance in HCC [19]. Abnormal methylation can silence tumor suppressor genes, ultimately promoting tumor progression and drug resistance. It may also affect the expression of DNA repair genes, contributing to therapeutic resistance [20]. Current evidence indicates that the TET1 protein, which is typically overexpressed in HCC and linked to drug resistance, impacts sorafenib resistance by modulating promoter methylation and regulating the expression of DNA repair–related genes [21]. These mechanisms may offer new perspectives for improving HCC management.

## 2. Materials and Methods

### 2.1. Data Collection and Preprocessing

TCGA (https://portal.gdc.cancer.gov/) was used to obtain Level 3 RNA‐seq data for liver hepatocellular carcinoma (LIHC), encompassing 50 nontumor and 374 tumor samples [22]. Furthermore, the Illumina Infinium HumanMethylation450 array was used to obtain DNA methylation data, encompassing 50 normal samples and 380 tumor samples, totaling 430 LIHC cases. To ensure data reliability, only patients with clinical follow‐up exceeding 30 days were included in the analysis. MDG and transcriptome matrices were extracted using a Perl script (5.30) for data merging and gene ID conversion (http://www.perl.org/). A beta value varying from 0 (*unmethylated*) to 1 (*fully methylated*) was used to represent the methylation level at each locus. To mitigate batch effects, MDG array data were integrated and adjusted via the ComBat algorithm from the sva package in R while retaining patient‐related biological variation. The GEO was applied to acquire the gene expression of the GSE10186 cohort.

Inclusion criteria were as follows:

1. Patients with histologically confirmed HCC.
2. Samples derived from the TCGA‐LIHC project and the GEO dataset (GSE10186).
3. Samples with both available DNA methylation data (e.g., 450K/850K array data) and RNA‐seq (or gene expression microarray) data.
4. Complete OS data, including survival status (alive/deceased) and exact survival time.

Exclusion criteria were as follows:

1. Duplicate samples from the same patient (only the first sample per patient retained).
2. Patients with a survival time of ≤ 30 days.
3. Samples with substantial missing values (missing rate > 20*%*) in either the DNA methylation or gene expression data matrix.

### 2.2. DEG Identification

mRNA expression data from GEO (118 tumor samples) and TCGA (380 tumor and 50 normal samples) were examined via the Limma package in R. One hundred and seventy‐eight tumor and 171 normal samples were included in the integrated analysis. DEGs, including both upregulated and downregulated genes in tumor tissues, were selected via the Limma package in R. DEGs were defined as those with |log2FC| > 2 and a false discovery rate (FDR) of < 0.05.

### 2.3. Screening of MDGs

Three data matrices were generated, including normal MDGs, gene expression profiles, and tumor MDGs. MDGs were obtained by comparing methylation profiles between tumor and normal tissues using the MethylMix package in R. Subsequently, correlation analyses were carried out to detect MDG events negatively correlated with gene expression among DEGs. A combined model was then constructed to evaluate the methylation status of genes. Finally, the differences in MDG levels between normal and tumor samples were assessed by the Wilcoxon rank sum test. The significance threshold was defined as *p* < 0.05, with a correlation coefficient cutoff set at < −0.3.

### 2.4. Survival Analysis

Kaplan–Meier (K‐M) survival analysis was carried out via the survminer software to determine optimal cutoff values for each dataset and analyze the association between MDGs and the OS among individuals with LIHC.

### 2.5. Construction of the Nomogram Signature

A nomogram signature was developed by integrating independent prognostic variables. The predictive performance of this nomogram was analyzed via calibration curves, with the 45° line indicating perfect concordance between predicted and observed outcomes.

### 2.6. Gene Set Enrichment Analysis (GSEA)

Functional differences were investigated between the low‐ and high‐risk groups through GSEA [23]. Statistical significance was set at a *p* value of < 0.05.

### 2.7. Drug Sensitivity Prediction

Chemotherapeutic drug sensitivity and tumor drug resistance were predicted using data from the Genomics of Drug Sensitivity in Cancer (GDSC) database (https://www.cancerrxgene.org/). The half‐maximal inhibitory concentration (IC_50_) values were estimated via the pRRophetic package in R.

### 2.8. Cell Transfection and CCK8 Assay

The SNU‐387 cell line, accessible at iCell (http://www.icellbioscience.com/search), was maintained in a DMEM medium containing 10% fetal bovine serum and 1% penicillin–streptomycin. They were incubated at 37°C in a 5% CO_2_ atmosphere within a humidified incubator. Subsequently, they were cultured in six‐well plates at 1 × 10^5^ cells/well and subjected to transfection using either negative control (NC) or si‐TEAD4/si‐CLGN/si‐GLS (GenePharma). The siRNA sequences were as follows: TEAD4‐Homo forward GGACAUCCGCCAAAUCUAUTT, reverse AUAGAUUUGGCGGAUGUCCTT; CLGN‐Homo forward GAGCAAAGCAUCAUGCAAUTT, reverse AUUGCAUGAUGCUUUGCUCTT; and GLS‐Homo forward CCACAUAAUCCUAUGGUAATT, reverse UUACCAUAGGAUUAUGUGGTT. After 6 h of incubation, cells in a healthy growth state were collected and adjusted to a specific concentration in suspension. Subsequently, 2 × 10^3^ cells in 100 *μ*L were added to each well of four individual 96‐well plates. CCK8 assays were carried out at 0, 24, 48, and 72 h.

## 3. Results

### 3.1. DEG Identification in HCC From the GTEx and TCGA Databases

**Figure 1:**
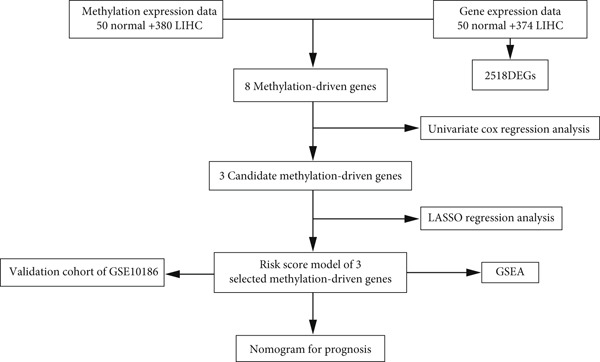
The flow diagram of the research procedure.

Figure 1 illustrates the analytical flowchart. RNA‐seq expression data, including 374 LIHC and 50 normal liver tissues, were collected from TCGA. Two thousand five hundred eighteen DEGs were identified after applying a filtering threshold of (|logFC| > 1, FDR 0.01). As illustrated in Figure 2a, 2090 DEGs were upregulated, while 428 were downregulated. The volcano plot is shown in Figure 2b.

Figure 2Identification of DNA methylation–driven genes and DEGs. (a) Heat map of the methylation levels of 100 candidate DNA methylation–driven genes in normal liver tissues (*n* = 50) and LIHC (*n* = 374). (b) Volcano plot of DEGs.

**Figure:**
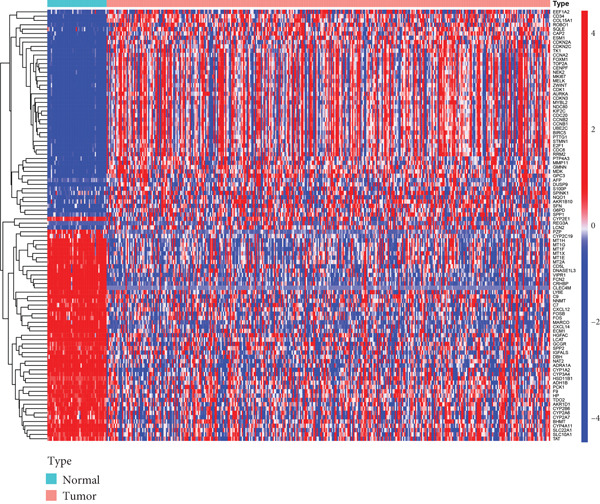
(a)

**Figure:**
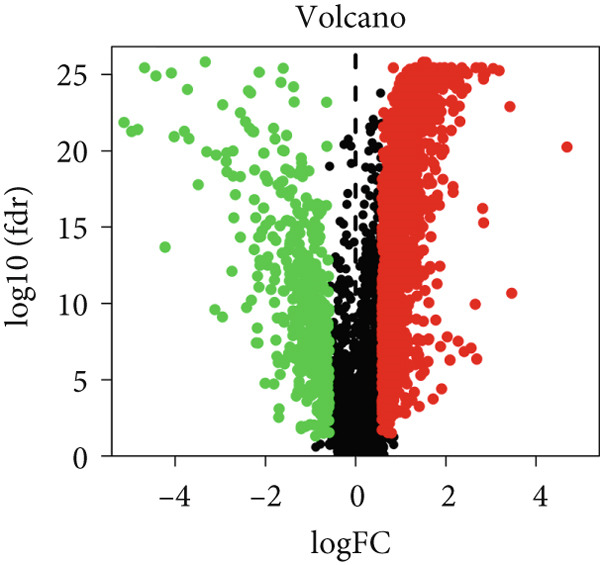
(b)

### 3.2. Identification of MDGs in LIHC

MethylMix analysis was carried out to obtain MDGs in LIHC. Genes with significantly altered methylation were selected according to a significance level of *p* < 0.05. Moreover, a correlation coefficient threshold below −0.3 between MDGs and mRNA expression was applied. This analysis identified eight MDGs. Among them, MT1E and KLF4 exhibited hypomethylation, while ZNF43, TEAD4, glutaminase (GLS), EFNB2, CLGN, and CCND2 were hypermethylated in LIHC. The heat map in Figure 3a,b illustrates the methylation levels of these MDGs. Figures 4a, 4b, 4c, 4d, 4e, 4f, 4g, and 4h present the methylation levels of these eight genes. In each graph, the histogram displays methylation distribution in the tumor group, while the curve suggests the estimated methylation trend in this group. Methylation levels in normal samples are marked by the green horizontal line, and those in tumor tissues are indicated in red. These graphs revealed that the eight genes demonstrated increased methylation levels in tumor samples than normal samples, indicating differences in methylation intensity distribution between the two groups. The relationship between methylation levels and gene expression for each gene was examined through Pearson′s correlation analyses (Figures 5a, 5b, 5c, 5d, 5e, 5f, 5g, and 5h). The *x*‐axis reflects the level of methylation, the *y*‐axis suggests gene expression, and the dashed line illustrates the relative trend. A negative association between methylation and gene expression was found for all eight genes.

Figure 3Identification of methylation‐driven genes using differentially expressed profiles. Hierarchical‐clustering heat maps of corresponding expression levels of (a) DEGs and (b) methylation status of 8 methylation‐driven DEGs.

**Figure:**
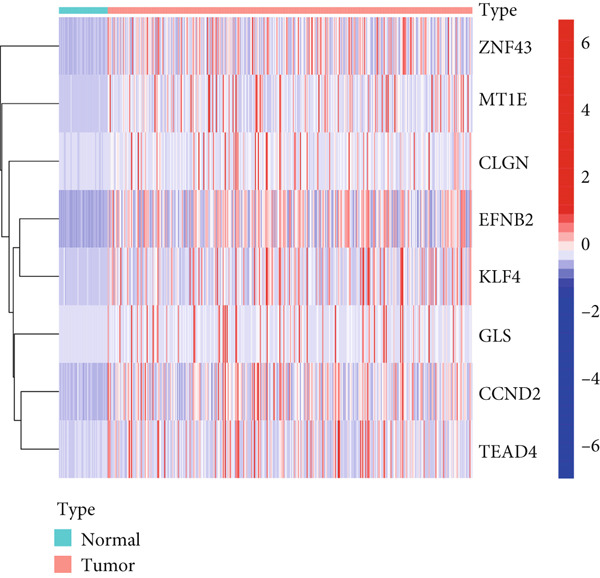
(a)

**Figure:**
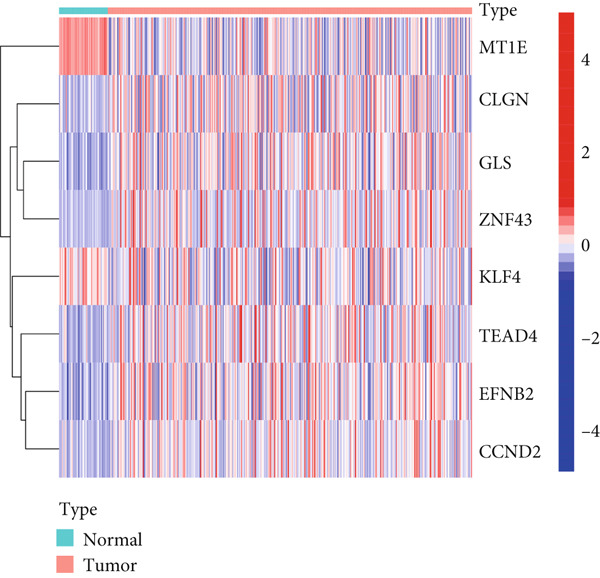
(b)

Figure 4Eight DNA methylation–driven genes identification employing MethylMix analysis. (a–h) Methylation statuses of eight DNA methylation–driven genes. The abscissa represents the methylation degree, the ordinate represents the quantity of methylation samples, the histogram depicts the methylation distribution of the cancer group, and the curve represents the predicted trend curve of the methylation distribution of the cancer group. The methylation level distribution of the normal sample is depicted by the dark horizontal line above the figure. The figure clearly displays the distribution of methylation degree in the cancer sample compared to the normal sample.

**Figure:**
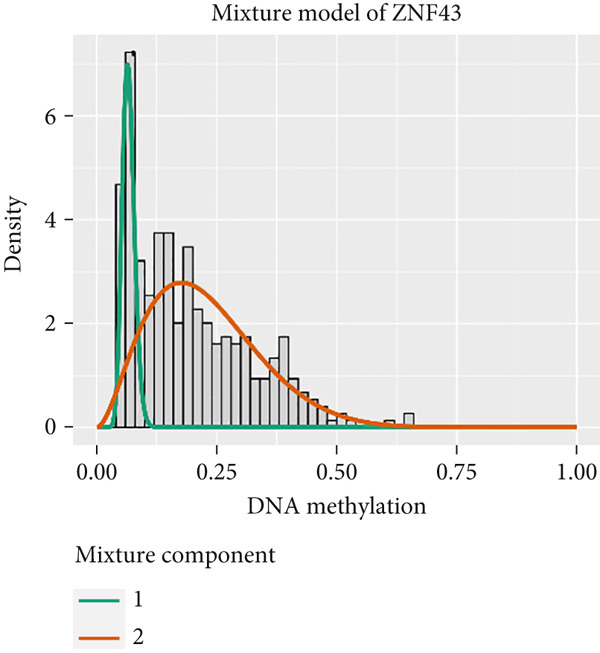
(a)

**Figure:**
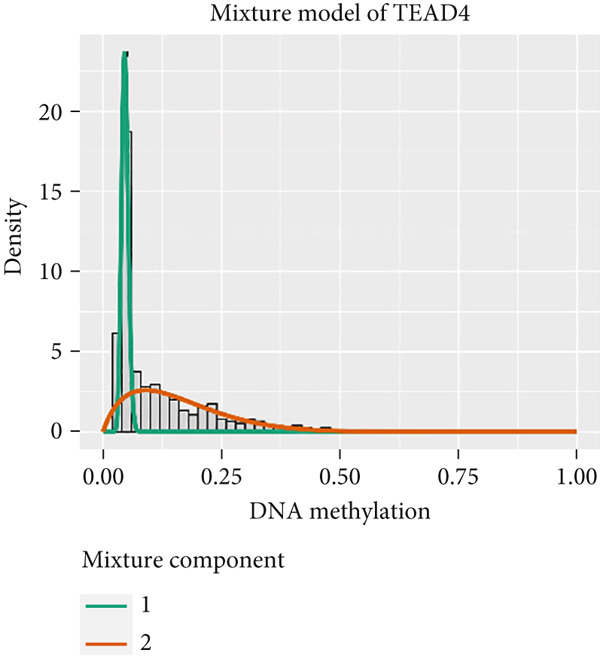
(b)

**Figure:**
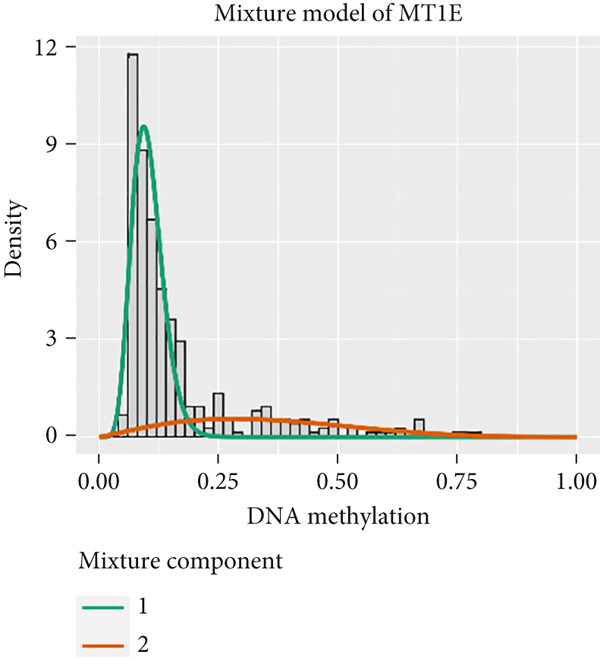
(c)

**Figure:**
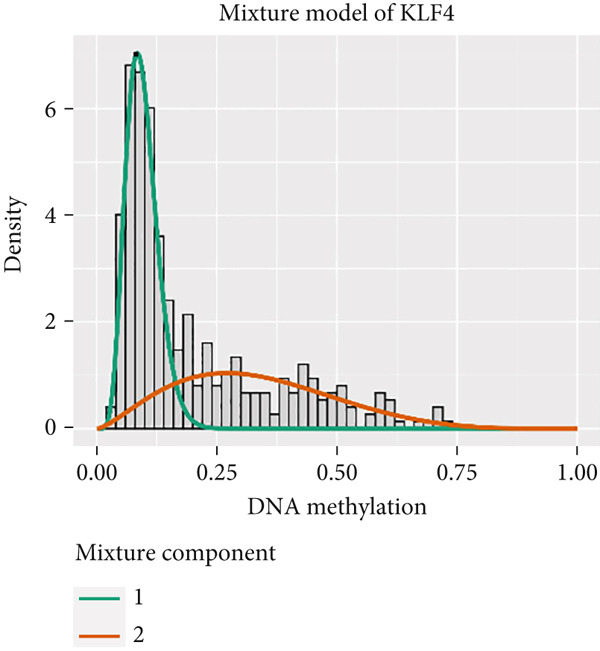
(d)

**Figure:**
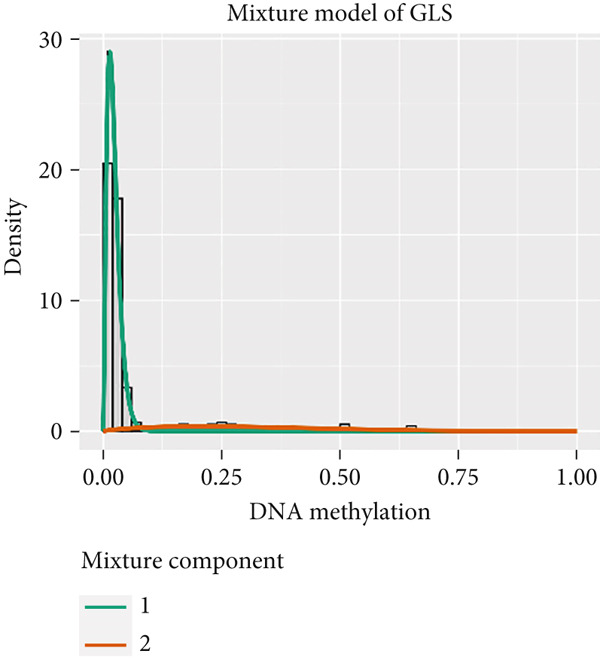
(e)

**Figure:**
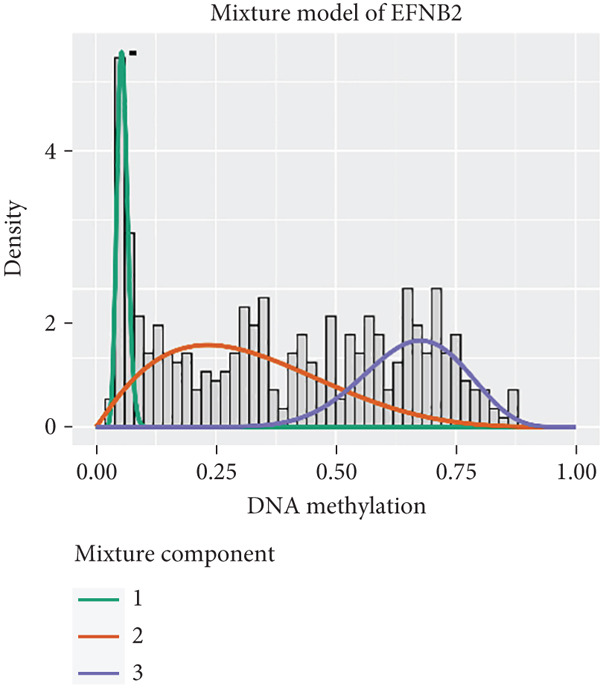
(f)

**Figure:**
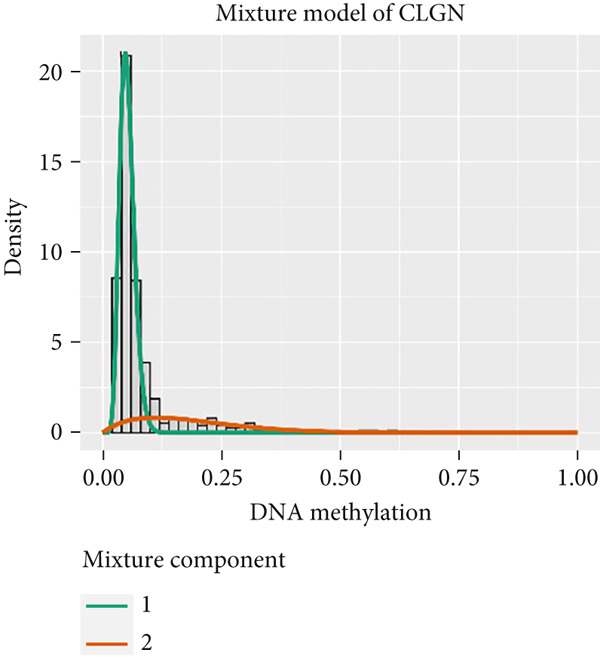
(g)

**Figure:**
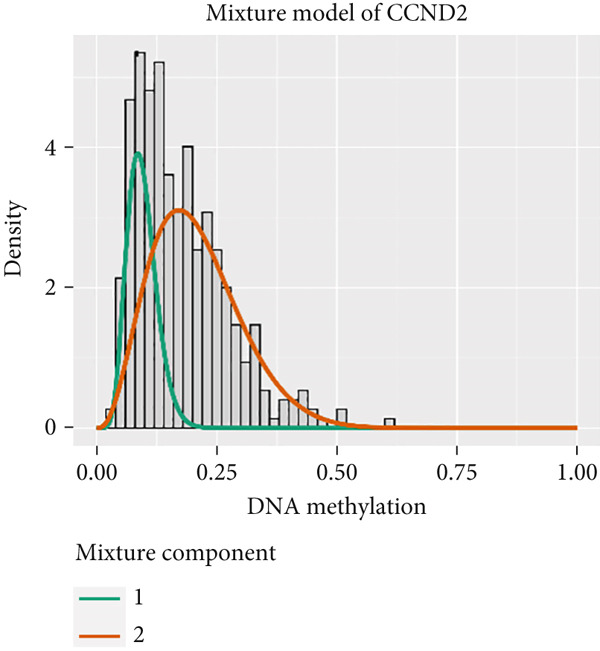
(h)

Figure 5Correlation analysis of the mRNA expression level and DNA methylation level in the eight MDGs. (a–h) The correlation between methylation level and gene expression was investigated using Pearson′s correlation analysis. The figure depicts that gene methylation is inversely linked to expression. The abscissa denotes gene methylation degree beta, and the ordinate denotes gene expression. Cor denotes the correlation coefficient, and *p* value denotes the correlation test value.

**Figure:**
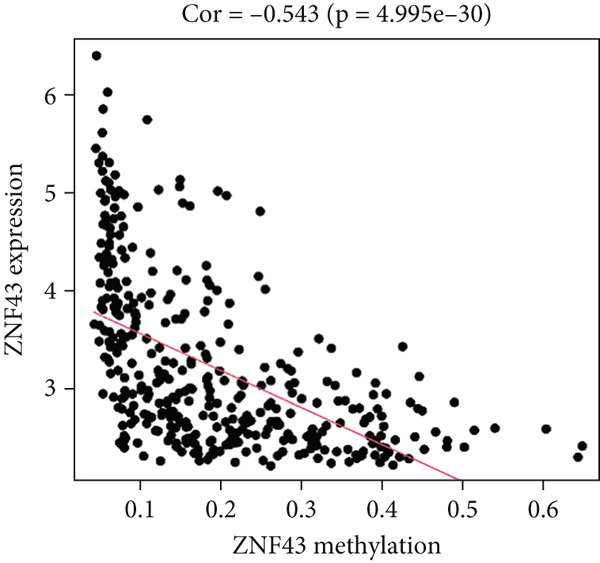
(a)

**Figure:**
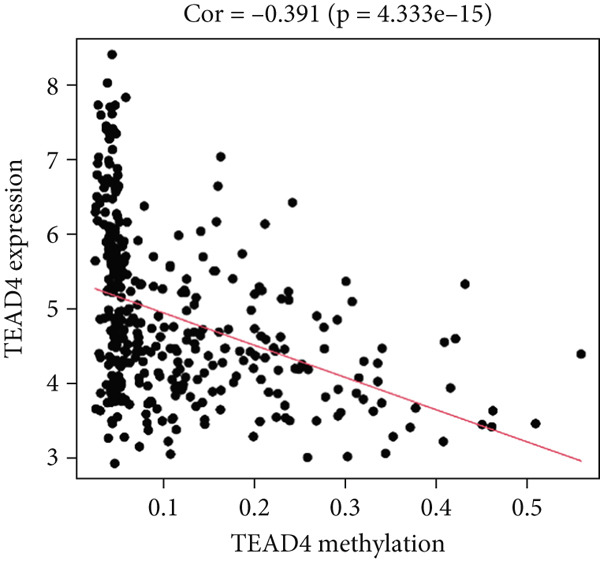
(b)

**Figure:**
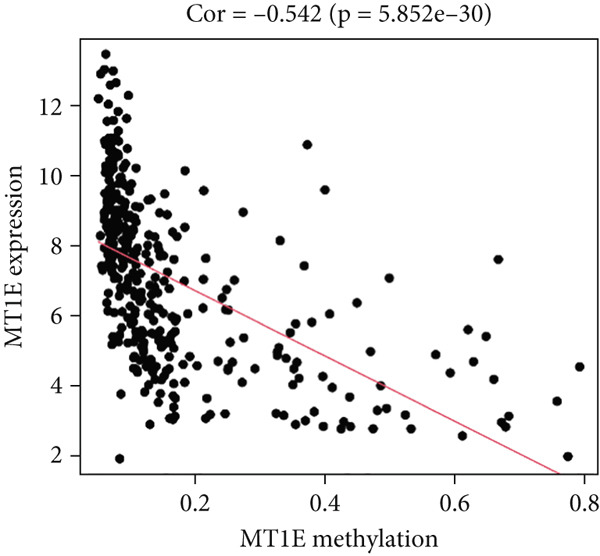
(c)

**Figure:**
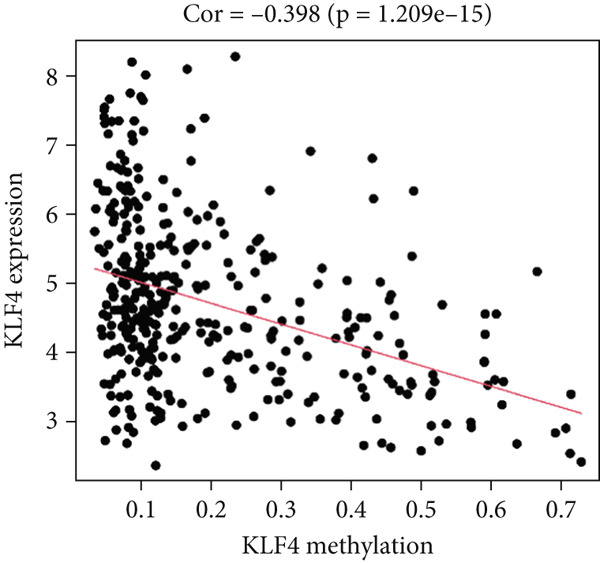
(d)

**Figure:**
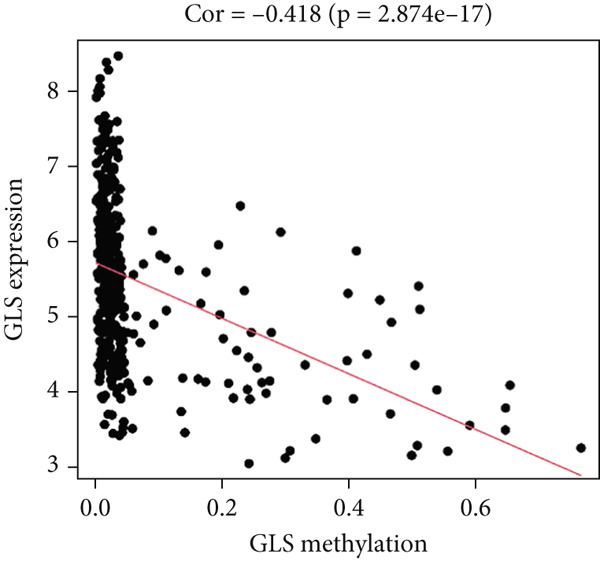
(e)

**Figure:**
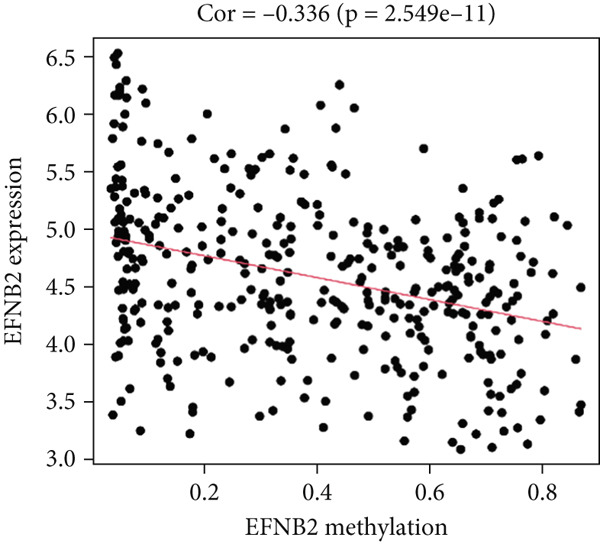
(f)

**Figure:**
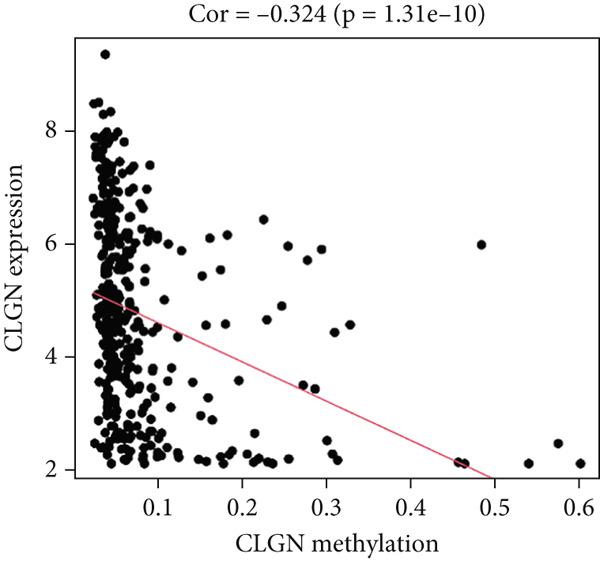
(g)

**Figure:**
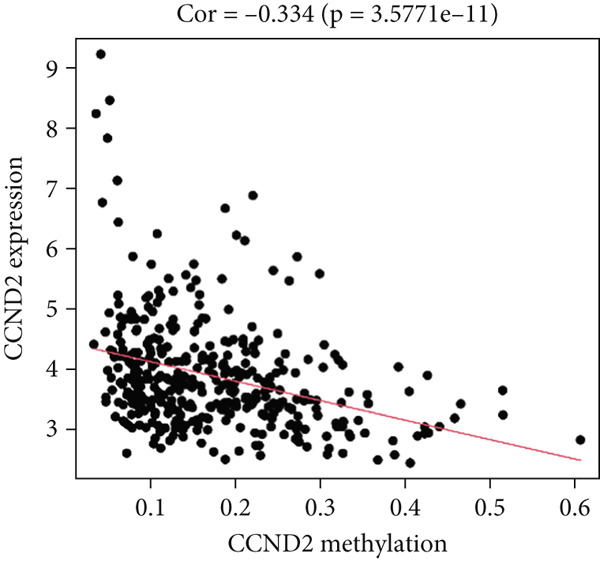
(h)

### 3.3. Construction of a Predictive Model Incorporating MDGs in LIHC

Univariable Cox proportional hazards regression analysis (Figure 6a) was carried out to examine the association between three candidate MDGs and OS (*p* < 0.05). Additionally, LASSO regression models were utilized to identify variables with nonzero coefficients by applying an L1 penalty that shrunk some regression coefficients toward 0 [24]. Moreover, after 1000 iterations of LASSO regression, variables with consistently nonzero coefficients were considered more reliable predictors of prognosis. Accordingly, three MDGs (GLS, TEAD4, and CLGN), which appeared in all iterations, were identified and incorporated into the risk score model (Figure 6b,c). The expression level of each gene was multiplied by its LASSO coefficient to calculate the risk score: 0.169 × GLS mRNA level + 0.094 × CLGN mRNA level + 0.145 × TEAD4 mRNA level. Using the median risk score (0.9534) as a cutoff, individuals with LIHC (TCGA cohort) were assigned into the low‐ or high‐risk groups. In terms of mRNA expression levels, the survival analysis (Figures 6d, 6e, and 6f) suggested that high‐risk patients demonstrated a worse OS (*p* < 0.001). Regarding methylation expression, the survival analysis (Figures 6g, 6h, and 6i) reported that high‐risk patients exhibited a longer OS (*p* < 0.001). In the TCGA cohort, survival analysis (Figure 7a) suggested that low‐risk patients had a markedly prolonged OS than the high‐risk patients (*p* = 0.002). The heat map and scatter plot (Figure 7e) were employed to visualize the expression profiles, survival status, and risk scores of MDGs for each HCC patient. ROC analysis was carried out to test the performance of the model for assessing OS. As illustrated in Figure 7c, the area under the curve (AUC) was 0.696 for the 1‐year OS, 0.600 for the 3‐year OS, and 0.594 for the 5‐year OS. For the validation cohort, 88 LIHC samples with complete survival data were collected from the GEO dataset (GSE10186). Applying the same median cutoff value and risk score formula, individuals were stratified into the high‐ and low‐risk groups. There was no evidence of a significant difference in OS between the two groups in the validation cohort (*p* = 0.224; Figure 7b). This result aligns with the earlier findings. Figure 7f presents a scatter plot of survival status and risk scores, alongside a heat map showing the expression of these three genes. When this model was applied to individuals with LIHC, the AUCs for 1‐, 3‐, and 5‐year OS were 0.597, 0.511, and 0.404, respectively (Figure 7d).

Figure 6DNA methylation–driven gene identification for predictive signature and corresponding survival analysis. (a) The univariable Cox regression method was utilized to determine three DNA methylation–driven genes. (b) The optimum amount of DNA methylation–driven genes was filtered using 1000 iterations of Cox LASSO regression with 10‐fold cross‐validation. (c) LASSO coefficients. (d–f) Survival analysis of model gene expression levels in low‐risk and high‐risk populations. (g–i) Survival analysis of model gene methylation levels in low‐risk and high‐risk populations.

**Figure:**
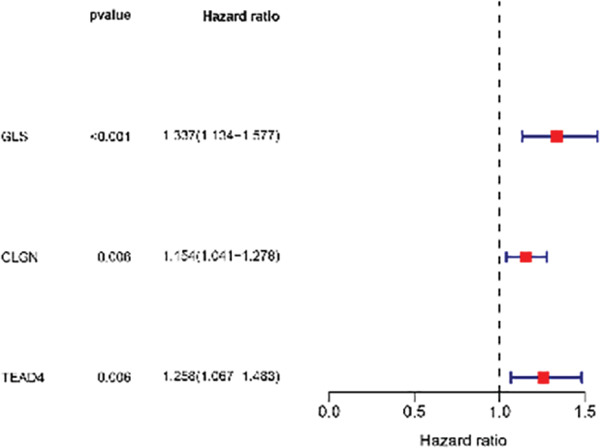
(a)

**Figure:**
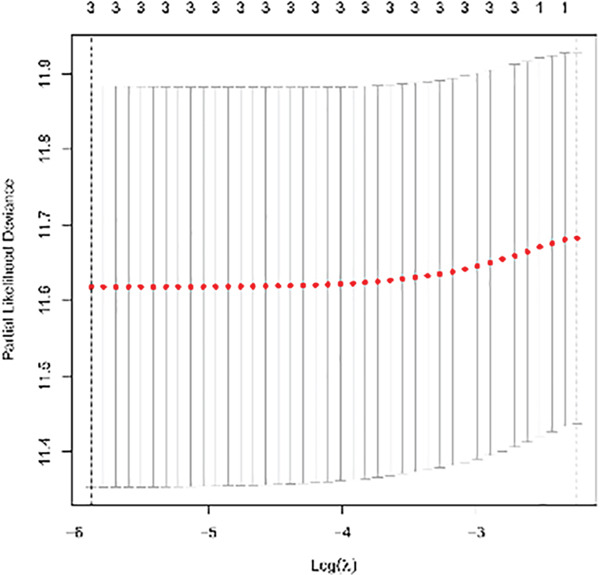
(b)

**Figure:**
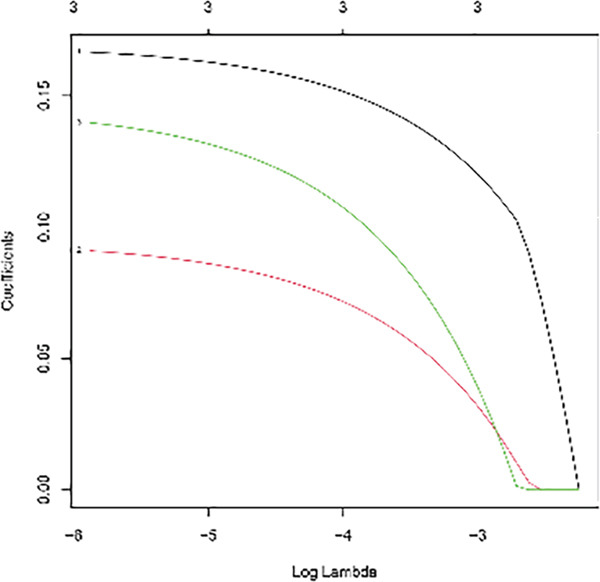
(c)

**Figure:**
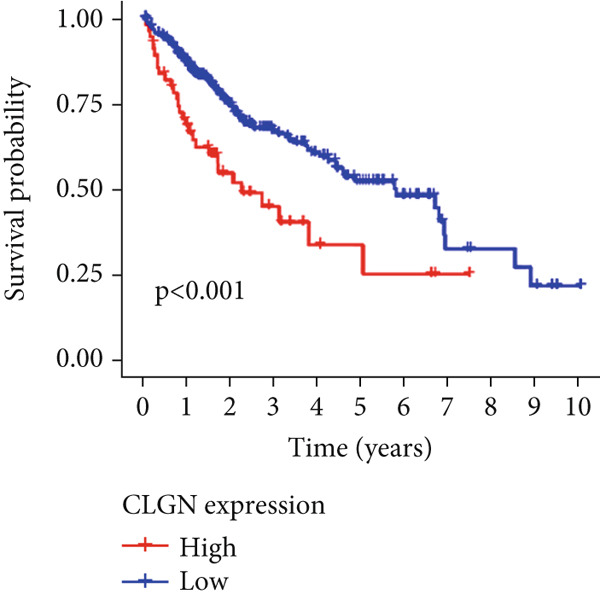
(d)

**Figure:**
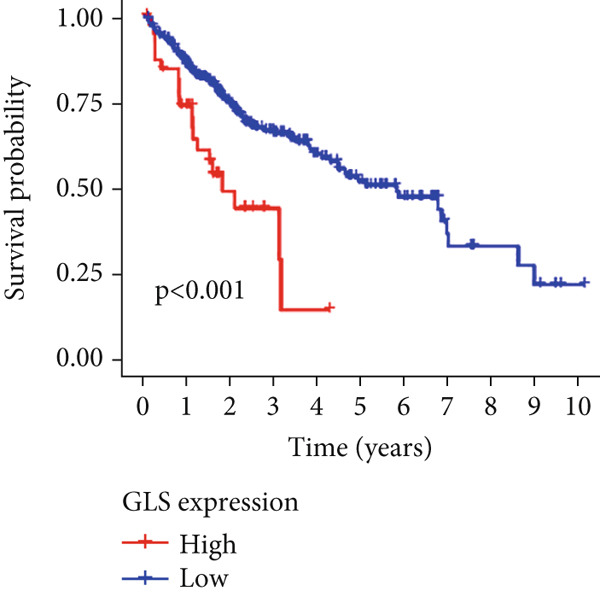
(e)

**Figure:**
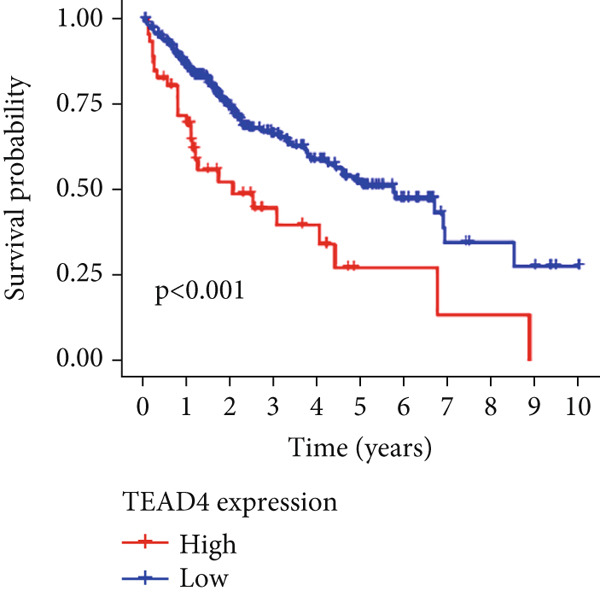
(f)

**Figure:**
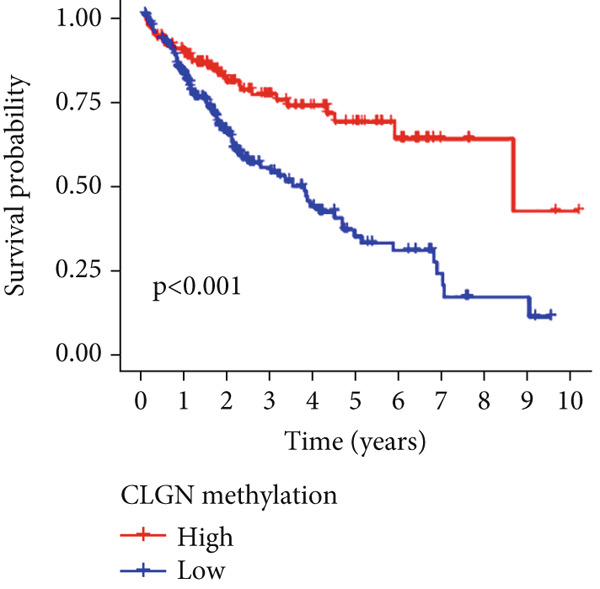
(g)

**Figure:**
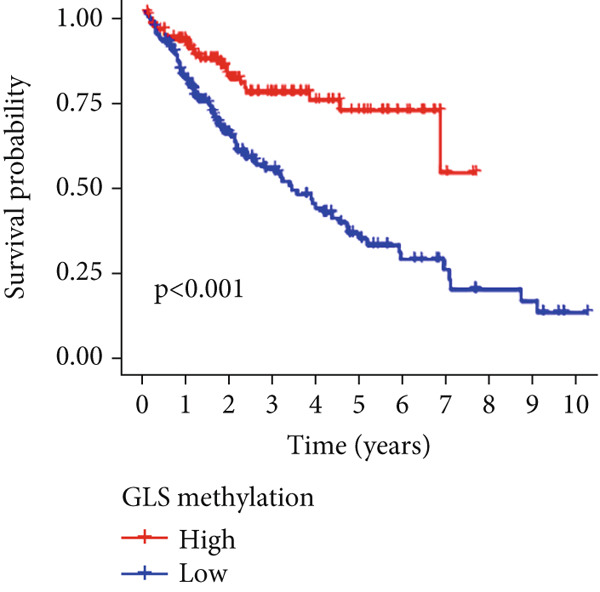
(h)

**Figure:**
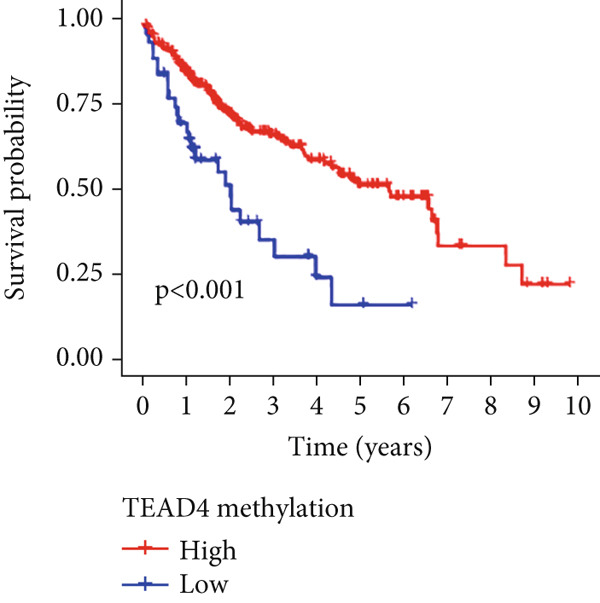
(i)

Figure 7(a) The overall survival (OS) K‐M curve for LIHC patients in two different groups in the TCGA cohort. (b) The K‐M curve of OS in the GEO cohort for LIHC patients is divided into two groups. (c) The time‐dependent ROC curve used to predict 1‐, 3‐, and 5‐year OS rates in the TCGA cohort. (d) The time‐dependent ROC curve used to predict 1‐, 3‐, and 5‐year OS rates in the GEO cohort. (e) The risk score distribution in LIHC patients, the survival status of LIHC patients, and the expression heat map of four methylation‐driven genes (MDGs) in the Cancer Genome Atlas (TCGA) cohort. (f) LIHC patient survival status, LIHC patient risk score distribution, and expression heat map of four MDGs in a GEO cohort.

**Figure:**
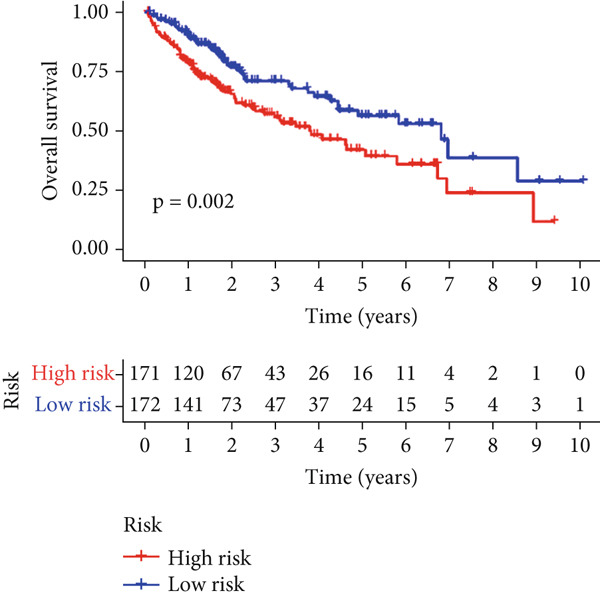
(a)

**Figure:**
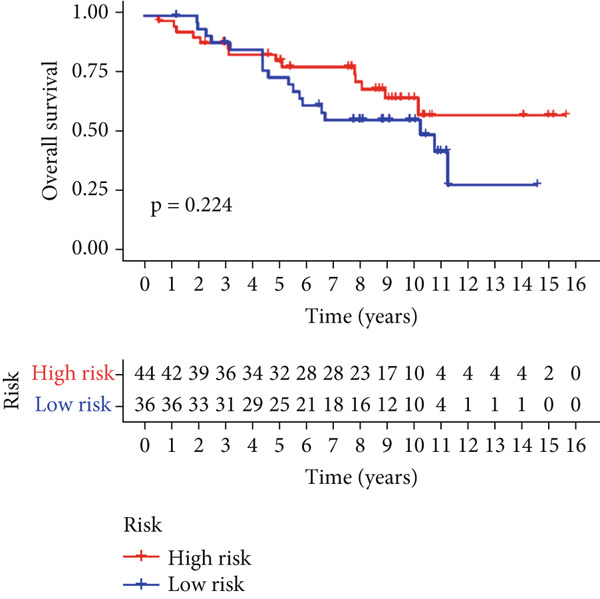
(b)

**Figure:**
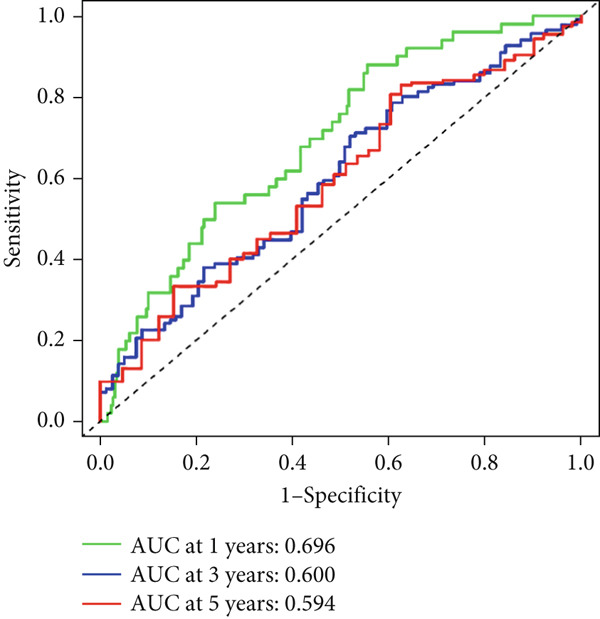
(c)

**Figure:**
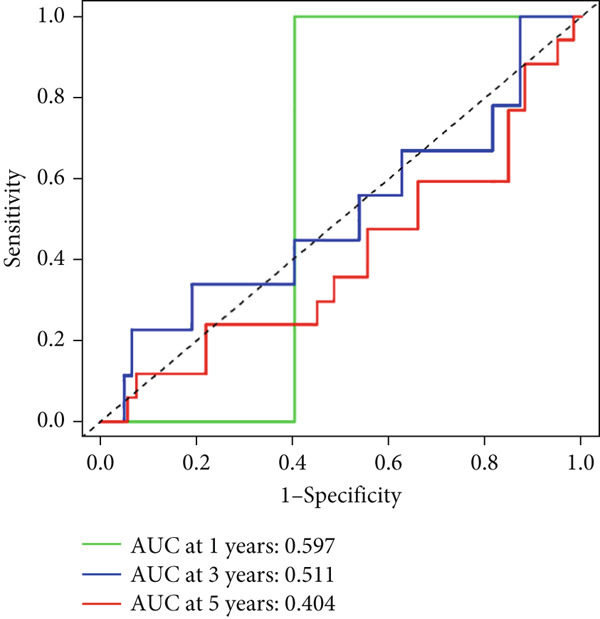
(d)

**Figure:**
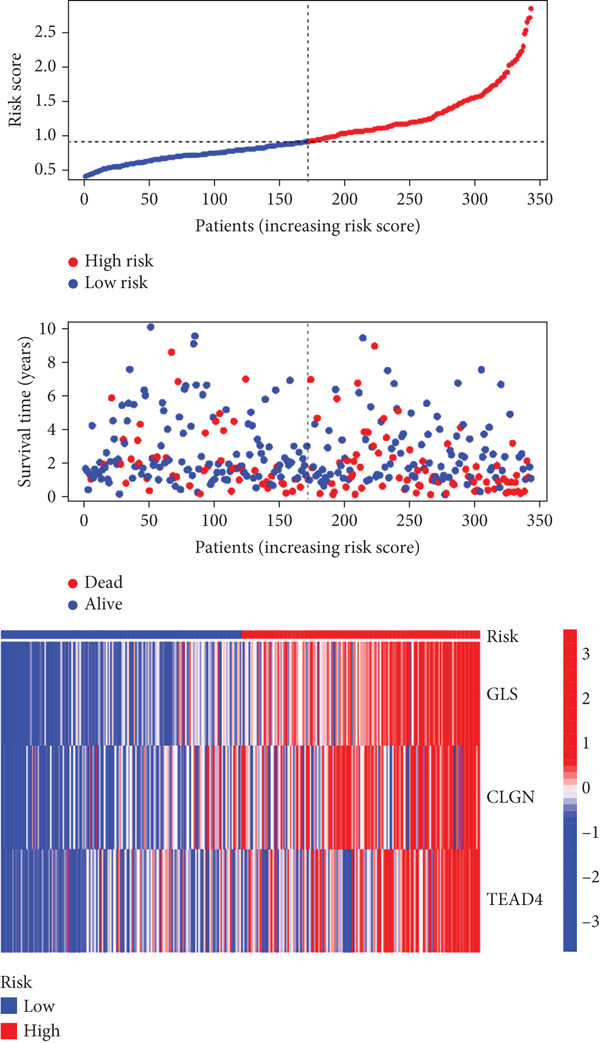
(e)

**Figure:**
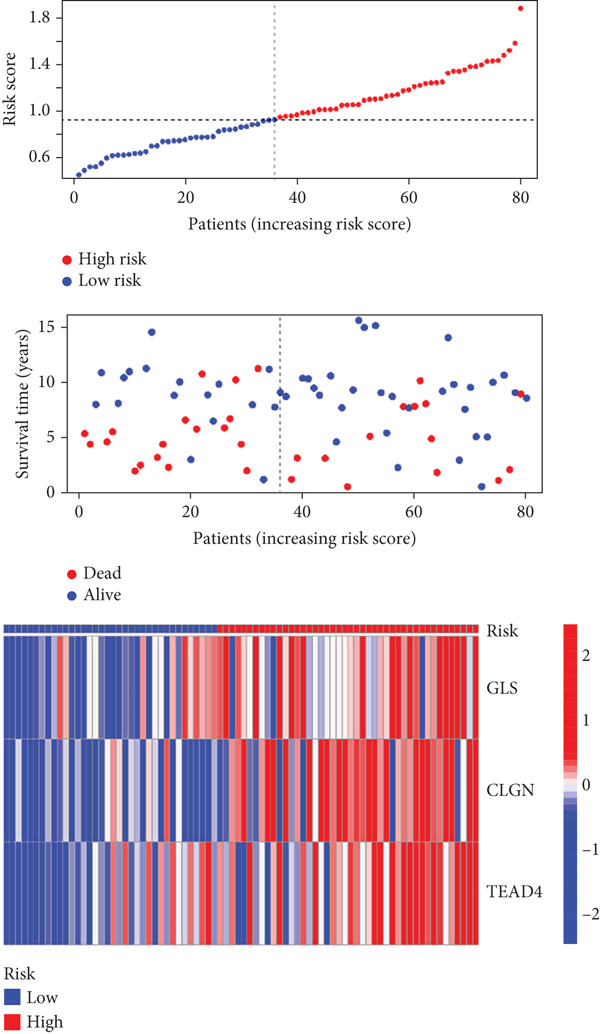
(f)

### 3.4. Independent Prognostic Value of the Risk Score

Multivariable and univariable Cox regression models were utilized to investigate the performance of this predictive model. These analyses were conducted in a cohort of 379 LIHC patients with comprehensive clinical data from TCGA, such as gender, stage, grade, and age. The univariable analysis revealed that stage and risk score were notably linked to OS. Multivariable analysis further confirmed these two factors as independent prognostic indicators (*p* < 0.05). In contrast, age, gender, and grade were not notably linked to OS in both models (Figure 8a,b).

Figure 8Nomo signature development and validation in the TCGA cohort. (a) Univariable and (b) multivariable Cox regression analyses of the clinical characteristics and risk score. (c) Nomo signature for predicting survival in patients with LIHC. (d) Calibration curves of 1‐, 3‐, and 5‐year OS.

**Figure:**
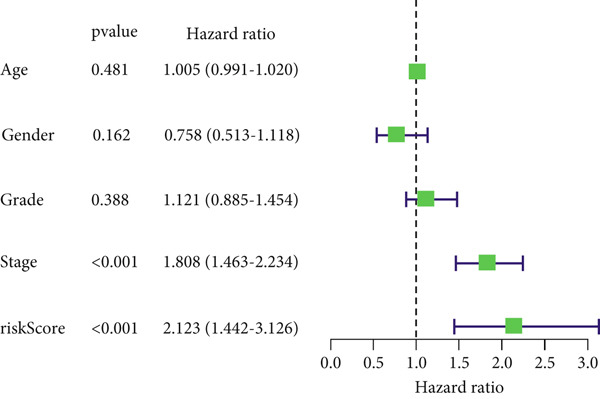
(a)

**Figure:**
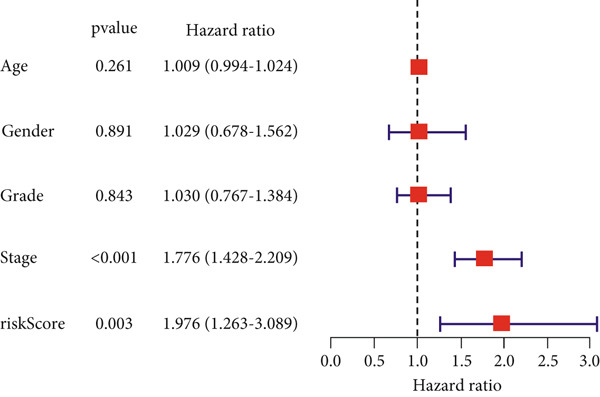
(b)

**Figure:**
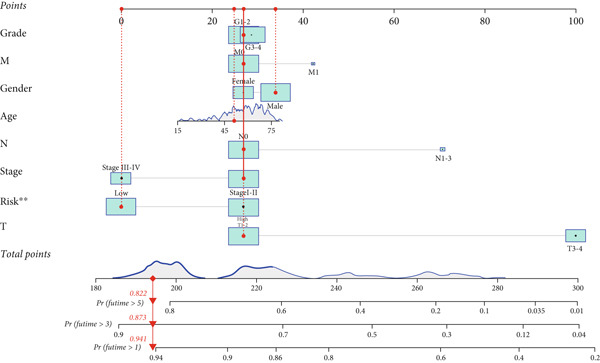
(c)

**Figure:**
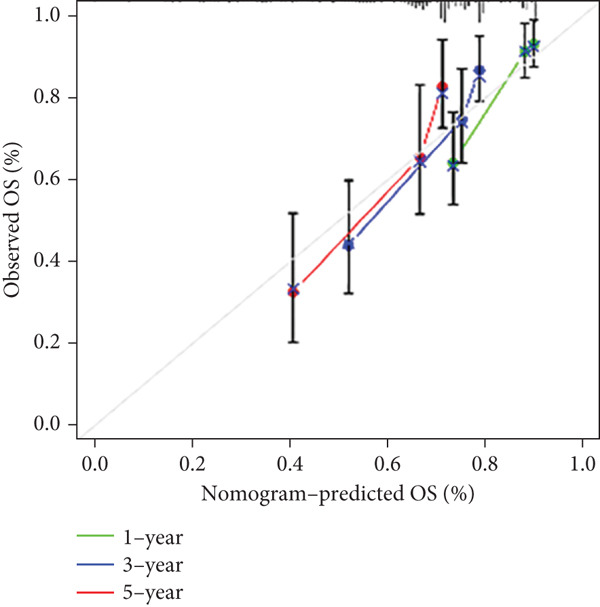
(d)

### 3.5. Construction and Evaluation of a Predictive Nomogram

A prognostic nomogram incorporating gender, stage, age, T, M, N, grade, and risk score derived from the signature was built for assessing OS. This nomogram effectively assessed 1‐, 3‐, and 5‐year OS (Figure 8c). Its performance was further evaluated via calibration curves, where ideal predictions align with the 45° line. As illustrated in Figure 8d, the nomogram exhibited reliable predictive performance for 1‐, 3‐, and 5‐year OS.

### 3.6. Drug Sensitivity Prediction and GSEA in the Low‐ and High‐Risk Groups

The molecular mechanisms underlying the risk in the high‐ and low‐risk groups were examined via GSEA. As illustrated in Figure 9a, the top five enriched pathways in the high‐risk group encompassed the following: cytokine–cytokine receptor interaction, ECM receptor interaction, focal adhesion, hematopoietic cell lineage, and neuroactive ligand receptor interaction. As illustrated in Figure 9b, the top five enriched pathways in the low‐risk group included the following: drug metabolism cytochrome P450, fatty acid metabolism, metabolism of xenobiotics by cytochrome P450, primary bile acid biosynthesis, and retinol metabolism. These enriched pathways revealed critical molecular targets and mechanisms related to LIHC. Subsequently, the GDSC database was utilized to assess the suitability of chemotherapy drugs for low‐ and high‐risk patients.

Figure 9The elevated KEGG pathways in the (a) high‐ and (b) low‐risk groups by GSEAs. (c) Drug sensitivity prediction of the risk signature in LIHC. Drug susceptibility analysis of therapy in the high‐ and low‐risk groups.

**Figure:**
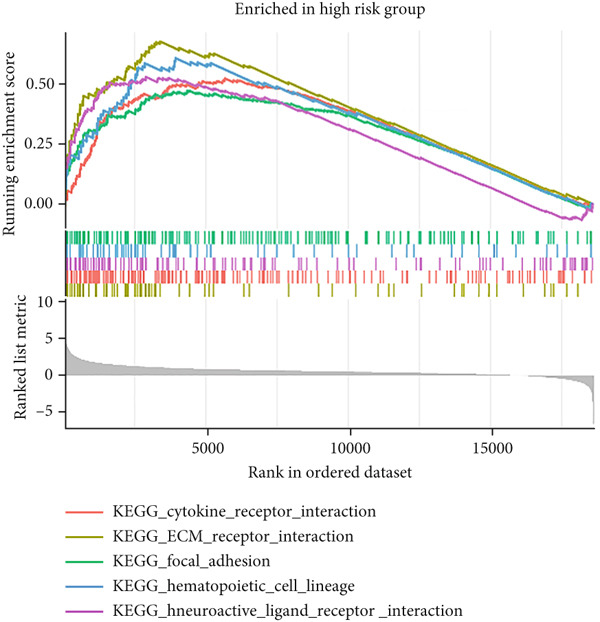
(a)

**Figure:**
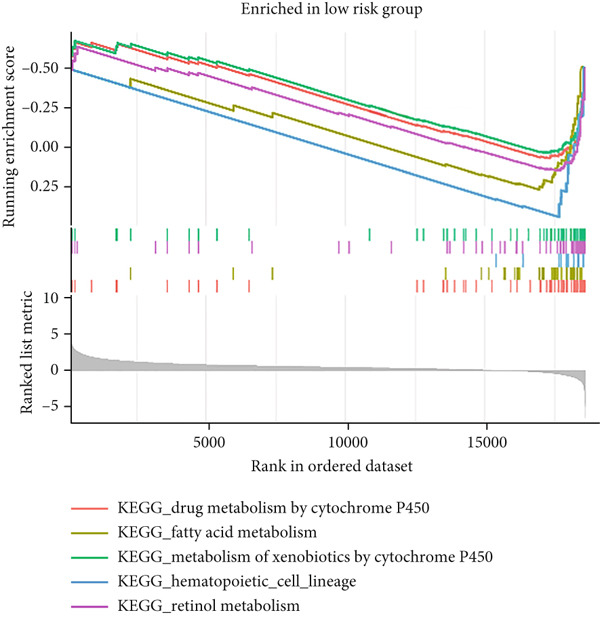
(b)

**Figure:**
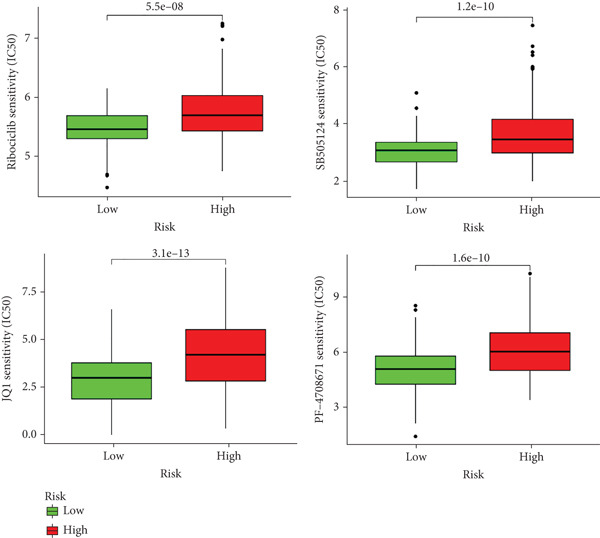
(c)

HCC patients at low risk demonstrated a favorable response to SB505124, ribociclib, JQ1, and PF4708671 (Figure 9c), as indicated by notably higher IC_50_ values in comparison to high‐risk patients (all *p* < 0.05). The pRRophetic packages were employed to evaluate the efficacy of antitumor drugs, including etoposide, vinorelbine, masitinib, bexarotene, midostaurin, 5‐fluorouracil (5‐FU), tipifarnib, and doxorubicin. The sensitivity of these medications was assessed. The results demonstrated that high‐risk patients exhibited reduced IC_50_ values, suggesting their greater sensitivity to these drugs (Figures S1–S83).

Finally, in vitro validation experiments were performed on the model genes CLGN, TEAD4, and GLS using SNU‐387 cells. The CCK8 assay, presented in both line chart (Figure 10a) and box plot formats (Figure 10b), demonstrated significantly reduced cell proliferation in the si‐CLGN, si‐TEAD4, and si‐GLS groups compared to the si‐NC group.

Figure 10In vitro validation on CLGN, TEAD4, and GLS in SNU‐387 cells. (a) Line chart of CCK8 assay. (b) Box plot of CCK8 assay.

**Figure:**
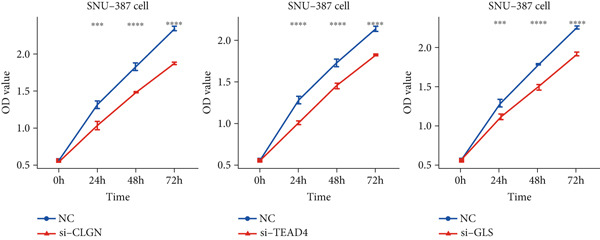
(a)

**Figure:**
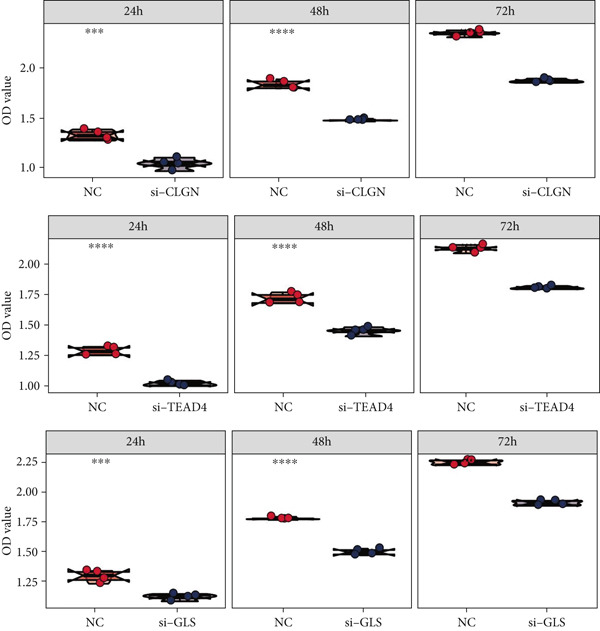
(b)

## 4. Discussion

HCC stands as one of the most lethal cancers, and its prognosis remains poor [25, 26]. Despite advances in current therapies for LC, such as surgical resection, chemotherapy, physiotherapy, and molecular targeted therapy, their effects on disease progression are limited [27–30]. The LC prognosis remains limited, with a 5‐year OS rate under 5% [31, 32]. Reliable methods for predicting outcomes in HCC patients are still lacking, representing a major global public health concern. Hence, identifying novel prognostic indicators and developing more accurate predictive models are of great importance. Over the past decade, molecular markers have demonstrated potential in predicting the prognosis of various tumors, with MDGs emerging as a prominent epigenetic feature in cancer biology [16–18]. In mammals, MDGs primarily occur within CpG islands, typically observed in the initial exon, the promoter region, and the 3 ^′^ end of genes [33]. Notably, nearly 70% of human gene promoters are located within CpG islands [34]. Existing evidence demonstrates that nearly all tumors exhibit abnormal MDGs compared to their corresponding nontumor normal tissues [35]. Therefore, MDGs may serve as a fundamental predictive biomarker [33, 34]. In recent years, numerous clinical and experimental studies have investigated gene methylation in the diagnosis, treatment, and prognosis of multiple malignant tumors, including breast cancer [36], lung cancer [37], and gastric cancer [38]. These findings indicate that MDGs significantly influence HCC.

HCC is marked by extensive genetic and epigenetic alterations [39]. An article by Liu et al. observed a notable correlation between M6A methylation–related gene expression and clinical features of HCC, indicating their potential as prognostic markers and drug targets [40]. Additionally, experimental data indicate that MDGs can regulate P14ARF mRNA levels in primary LC, with P14ARF MDGs possibly linked to HCC incidence and TNM stage [41]. Moreover, an article by Ng et al. [42] found notable methylation of NKAPL in LC, with methylation levels inversely linked to gene expression. This epigenetic alteration was also linked to the suppressed proliferation of LC cells. In comparison to gene mutations, epigenetic alterations, particularly DNA methylation and histone modification, are potentially reversible [43]. Compounds including 5‐aza‐2 ^′^‐deoxycytidine and 5‐azacytidine reactivate the expression of genes associated with MDGs in cancer cell lines. These compounds have demonstrated therapeutic efficacy in hematological malignancies and, in some cases, even solid tumors [44]. Hence, identifying MDGs for HCC is of critical importance.

This study performed an integrative analysis of RNA‐seq data and MDG profiles to explore genome‐wide MDG patterns in HCC. Initially, 100 MDGs were identified using the MethylMix algorithm by screening 2518 DEGs between normal and HCC tissues from the GTEx and TCGA datasets. Subsequently, univariable Cox and LASSO regression models were employed to identify three MDGs (GLS, TEAD4, and CLGN) notably associated with OS. These three genes are related to multiple cancers. For example, cancer cells reprogram glucose and glutamine (GLU) metabolism to sustain growth, and GLS significantly contributes to tumor progression [45]. The gene expression of GLS is positively linked to the expression of multiple immune cell indicators and immune cell infiltration, thereby impacting the prognosis of glioma patients [31]. In LC, GLS expression shows a negative correlation with miR‐122, suggesting that GLS may influence tumor progression by regulating GLU metabolism [46]. Moreover, enhanced GLU metabolism is a hallmark of aggressive malignancies, including triple‐negative breast cancer. GLS catalyzes the conversion of GLU to glutamate, which serves as the initial and rate‐limiting step of GLU metabolism. This enzyme is also a potential candidate for anticancer drug development [32]. A recent study [47] demonstrated that HCC patients with higher levels of calmegin (CLGN) mRNA exhibited shorter disease‐free survival (DFS), disease‐specific survival (DSS), progression‐free survival (PFS), and OS. Furthermore, TEAD4 significantly impacts embryonic growth and tumor formation [48]. Elevated TEAD4 expression is observed in GI cancer tissues, and its CpG site is significantly hypomethylated. This reduced methylation of the TEAD4 promoter correlates with unfavorable predictive factors, such as larger tumor size, higher tumor grade, and decreased survival rates [49]. The study also found that TEAD4 promotes cell proliferation and tumor growth by directly regulating the expression of HSP70 family members, a mechanism independent of YAP and TAZ, indicating that TEAD4 exerts a Hippo‐independent function in HCC [50]. Research by Dugué et al. discovered that a methylation marker in CLGN is linked to the incidence of prostate cancer [51]. Recent studies demonstrate that mRNA levels of CLGN are significantly upregulated in HCC. Individuals with high CLGN expression exhibit poorer OS, DFS, PFS, and DSS. Further analyses have indicated that elevated expression of CLGN mRNA in the advanced pathological stage is linked to worse prognosis [47].

By incorporating these three MDGs, a risk score model was established to assess the prognosis for HCC. According to the risk scores, individuals were assigned to a high‐ or low‐risk group. Notably, in the TCGA training dataset and the GEO validation cohort (GSE10186), high‐risk patients exhibited shorter OS in comparison to low‐risk patients. Its accuracy was confirmed using time‐dependent AUC values for 1‐, 3‐, and 5‐year OS. The results demonstrated a robust performance. Additionally, clinical factors (age, gender, stage, risk score, grade, T, N, and M) were evaluated as independent prognostic indicators for OS. Subsequently, a nomogram signature integrating the risk score and clinical variables was developed to assess individual survival probability in clinical settings. Calibration curves revealed that this nomogram demonstrated robust and reliable prognostic performance. The practical value of the nomogram in clinical practice has been validated and applied across multiple medical fields. By integrating clinical data and imaging characteristics, the nomogram provides personalized risk assessment and prognosis prediction, thereby assisting clinicians in formulating more precise treatment strategies. First, in breast cancer patients, the nomogram is used to predict the risk of postoperative flap necrosis. Studies indicate that the nomogram model, constructed by analyzing nine independent risk factors including age, body mass index, and neutrophil count, achieved an AUC of 0.898 in the training cohort and 0.886 in the validation cohort, demonstrating excellent discriminatory and calibration capabilities. This model offers significant clinical benefits, aiding risk management and improving patient outcomes [52]. Furthermore, the nomogram has demonstrated value in predicting postoperative recurrence of nonfunctional large pituitary adenomas. By integrating clinical data and imaging features, researchers developed a comprehensive model achieving an AUC of 0.863 on an external validation set, indicating high diagnostic performance and clinical utility. This model can guide postoperative follow‐up strategies and facilitate early adjuvant therapy for high‐risk patients [53]. Key biological mechanisms underlying different risk groups were investigated through GSEA. The following pathways were notably enriched in the high‐risk group: cytokine receptor interaction, focal adhesion, hematopoietic cell lineage, neuroactive ligand receptor interaction, and ECM receptor interaction. In contrast, the mainly enriched pathways in the low‐risk score group were as follows: primary bile acid biosynthesis, retinol metabolism, metabolism of xenobiotics by cytochrome P450, fatty acid metabolism, and drug metabolism by cytochrome P450.

Chemoresistance remains a major challenge in HCC and is linked to poor prognosis. Our findings reveal that low‐risk patients might benefit from a combination therapy involving drugs such as dasatinib, midostaurin, bicalutamide, and docetaxel. It suggests that this risk model incorporating MDGs may serve as a valuable tool for guiding chemotherapy strategies in HCC.

Research by Long et al. developed and validated a predictive, recurrence, and diagnostic model incorporating two MDGs for HCC [54]. To our knowledge, our model may be the first to incorporate three MDGs in HCC. Our study further validated the effects of these three genes on HCC cells in vitro using the SNU‐387 cell line. Furthermore, their potential chemotherapeutic sensitivities across different risk groups were predicted by integrating GSEA and GDSC databases. These findings may aid clinical prognosis evaluation and inform therapeutic strategies for individuals with HCC. Nevertheless, this study has certain limitations. Although prior studies have linked the methylation or expression of these signature genes to cancer outcomes, the specific roles of these three genes in HCC remain unclear. Additionally, although the risk score model demonstrated strong performance in the TCGA and external validation cohorts, evidence supporting its superiority over conventional diagnostic tools such as imaging or alpha‐fetoprotein testing remains insufficient. Further biological investigations are thus required to elucidate the specific impacts of these three genes on HCC. Moreover, although this nomogram, which integrated clinical characteristics and risk scores, demonstrated reliable predictive accuracy, its generalizability remains limited due to the restricted clinical data and lack of patient diversity. Although the prognostic model constructed in this study demonstrated good discriminatory ability in the TCGA training cohort, its performance significantly declined in the independent GEO validation cohort, with AUC values ranging from 0.404 to 0.597. We acknowledge this finding and consider an in‐depth analysis of its potential causes to be crucial. First, substantial heterogeneity between cohorts likely constitutes the primary factor undermining the model′s generalization capability. The TCGA and GEO datasets may originate from populations with differing etiological backgrounds (e.g., HBV‐predominant vs. HCV/NAFLD‐predominant cohorts), disease staging, treatment regimens, and follow‐up strategies. These variations in clinical and pathological characteristics directly influence the weighting of prognostic drivers, thereby limiting the universality of models trained on a single cohort. Second, technical batch effects cannot be overlooked. Despite standardizing DNA methylation and gene expression data from different platforms, residual technical biases may introduce noise that disrupts the stability of model features, leading to diminished predictive performance. Most importantly, this observation highlights the complexity of HCC prognostic mechanisms. The decline in model performance suggests that the prognostic value of the identified methylation‐driven genes (GLS, TEAD4, and CLGN) may be modulated to some extent by specific populations or molecular contexts. This does not entirely negate the model′s value but positions it as a significant exploratory finding. It reveals the role of these genes within specific contexts and underscores the necessity of considering applicability boundaries and utilizing multicenter, prospective cohorts for further optimization and validation before advancing biomarker‐based models into clinical application. Therefore, future multicenter studies with more diverse and larger cohorts are needed to refine and validate the model.

Methylation, an epigenetic mechanism that involves the addition of a methyl group to DNA, modulates gene expression by regulating chromatin accessibility to the transcriptional machinery. Aberrant MDG patterns were observed in LC, and these patterns are implicated in resistance to certain anticancer agents. HCC, a complex disease driven by diverse genetic and epigenetic alterations, exhibits dynamic changes in MDG patterns throughout its initiation and progression. These alterations notably impact the expression levels of genes linked to essential cellular processes, including apoptosis, drug metabolism, DNA repair, and cell cycle regulation. Aberrant DNA methylation may promote drug resistance by silencing genes that are essential for drug efficacy. For instance, promoter hypermethylation may suppress the expression of drug‐metabolizing enzymes (e.g., cytochrome P450), thereby impairing the role of LC cells to metabolize and eliminate certain drugs, ultimately reducing drug efficacy and promoting resistance.

The role of DNA methylation in LC drug resistance is a complex and multifaceted process. Research indicates that DNA methylation can promote resistance to chemotherapy drugs in LC cells through multiple mechanisms. First, DNA methylation can enhance resistance by inducing epithelial–mesenchymal transition (EMT). EMT is a cellular transformation process where epithelial cells acquire mesenchymal characteristics, thereby enhancing cell migration and invasion capabilities. This process is closely associated with drug resistance in multiple cancers [55]. In HCC, DNA methylation–driven EMT is recognized as a common mechanism for developing resistance to therapeutic agents like sorafenib [55]. Additionally, DNA methylation influences HCC drug resistance by regulating the expression of specific genes. For instance, studies reveal that HDAC11 overexpression correlates with enhanced sorafenib resistance and metastatic capacity in HCC, and this overexpression is associated with hypomethylation in its promoter region [56]. Using DNA methyltransferase inhibitors can reduce HDAC11 expression, thereby restoring sorafenib sensitivity [56]. Protein methylation is another critical factor in LC drug resistance. Protein methylation is a common posttranslational modification involved in diverse biological processes, including signal transduction, transcriptional regulation, and DNA repair. Studies indicate that protein methylation plays a pivotal role in the resistance of HCC cells to 5‐FU. By comparing the methylome profiles of resistant and sensitive cell lines, significant alterations at specific methylation sites were identified, potentially contributing to the development of resistance [57]. In summary, DNA methylation promotes drug resistance in HCC through multiple pathways, including inducing EMT, regulating key gene expression, and influencing protein methylation status. These findings not only deepen our understanding of the mechanisms underlying drug resistance in HCC but also provide potential targets for developing novel therapeutic strategies. Future research could further explore how to reverse drug resistance by modulating DNA methylation, thereby enhancing the therapeutic efficacy of HCC treatments.

## 5. Conclusions

Using data from the TCGA database, a predictive model incorporating three genes driven by MDGs was developed and integrated into a nomogram signature. This model demonstrated strong predictive power for HCC. By combining the patient age, tumor stage, gender, grade, and TNM classification with the risk score, this nomogram may provide personalized predictions of OS and assess drug resistance in HCC patients.

**Nomenclature**
HCC: hepatocellular carcinoma
TCGA: The Cancer Genome Atlas
LC: liver cancer
GEO: Gene Expression Omnibus
AUC: area under the curve
FC: fold change
FDR: false discovery rate
OS: overall survival
MDGs: DNA methylation
GLS: glutaminase
TEAD4: TEA Domain Transcription Factor 4
CLGN: calmegin
RIG‐I: retinoic acid–inducible gene I

## Disclosure

The final manuscript was reviewed and approved by all authors.

## Conflicts of Interest

The authors declare no conflicts of interest.

## Author Contributions

G.H. analyzed the data and wrote the manuscript. L.Z. designed the study and secured funding. J.Z. implemented the provided guidance and prepared the dataset. G.H. and L.Z. contributed equally to this work and should be considered as cofirst authors.

## Funding

The study was funded by the investigation of the role and mechanism of LincRNA‐00844 in regulating AZGP1 structure and function during the progression of liver cancer, 2021KY1101, and the application and assessment of a multidisciplinary fast‐track surgery concept in laparoscopic liver surgery, 2019AD32208, and the Application of magnetically controlled microarrays based on multi‐fluorescent pixel counting in predicting the risk of acute lung injury in severe acute pancreatitis, KLY26H200009.

## Supporting information


**Supporting Information** Additional supporting information can be found online in the Supporting Information section. Figure S1: IC_50_ estimation of chemotherapeutic efficacy in the high‐risk and low‐risk groups in the chemotherapeutic drug IWP. Figure S2: IC_50_ estimation of chemotherapeutic efficacy in the high‐risk and low‐risk groups in the chemotherapeutic drug taselisib. Figure S3: IC_50_ estimation of chemotherapeutic efficacy in the high‐risk and low‐risk groups in the chemotherapeutic drug telomerase. Figure S4: IC_50_ estimation of chemotherapeutic efficacy in the high‐risk and low‐risk groups in the chemotherapeutic drug temozolomide. Figure S5: IC_50_ estimation of chemotherapeutic efficacy in the high‐risk and low‐risk groups in the chemotherapeutic drug trametinib. Figure S6: IC_50_ estimation of chemotherapeutic efficacy in the high‐risk and low‐risk groups in the chemotherapeutic drug ulixertinib. Figure S7: IC_50_ estimation of chemotherapeutic efficacy in the high‐risk and low‐risk groups in the chemotherapeutic drug ULK1‐4989. Figure S8: IC_50_ estimation of chemotherapeutic efficacy in the high‐risk and low‐risk groups in the chemotherapeutic drug UMI‐77. Figure S9: IC_50_ estimation of chemotherapeutic efficacy in the high‐risk and low‐risk groups in the chemotherapeutic drug VE821. Figure S10: IC_50_ estimation of chemotherapeutic efficacy in the high‐risk and low‐risk groups in the chemotherapeutic drug VE‐822. Figure S11: IC_50_ estimation of chemotherapeutic efficacy in the high‐risk and low‐risk groups in the chemotherapeutic drug venetoclax. Figure S12: IC_50_ estimation of chemotherapeutic efficacy in the high‐risk and low‐risk groups in the chemotherapeutic drug vinblastine. Figure S13: IC_50_ estimation of chemotherapeutic efficacy in the high‐risk and low‐risk groups in the chemotherapeutic drug vinorelbine. Figure S14: IC_50_ estimation of chemotherapeutic efficacy in the high‐risk and low‐risk groups in the chemotherapeutic drug VX‐11e. Figure S15: IC_50_ estimation of chemotherapeutic efficacy in the high‐risk and low‐risk groups in the chemotherapeutic drug Wee1 inhibitor. Figure S16: IC_50_ estimation of chemotherapeutic efficacy in the high‐risk and low‐risk groups in the chemotherapeutic drug WEHI‐539. Figure S17: IC_50_ estimation of chemotherapeutic efficacy in the high‐risk and low‐risk groups in the chemotherapeutic drug WIKI4. Figure S18: IC_50_ estimation of chemotherapeutic efficacy in the high‐risk and low‐risk groups in the chemotherapeutic drug YK‐4‐279. Figure S19: IC_50_ estimation of chemotherapeutic efficacy in the high‐risk and low‐risk groups in the chemotherapeutic drug zoledronate. Figure S20: IC_50_ estimation of chemotherapeutic efficacy in the high‐risk and low‐risk groups in the chemotherapeutic drug JAK1‐8709. Figure S21: IC_50_ estimation of chemotherapeutic efficacy in the high‐risk and low‐risk groups in the chemotherapeutic drug staurosporine. Figure S22: IC_50_ estimation of chemotherapeutic efficacy in the high‐risk and low‐risk groups in the chemotherapeutic drug JQ1. Figure S23: IC_50_ estimation of chemotherapeutic efficacy in the high‐risk and low‐risk groups in the chemotherapeutic drug MIM1. Figure S24: IC_50_ estimation of chemotherapeutic efficacy in the high‐risk and low‐risk groups in the chemotherapeutic drug MIRA‐1. Figure S25: IC_50_ estimation of chemotherapeutic efficacy in the high‐risk and low‐risk groups in the chemotherapeutic drug MK‐1775. Figure S26: IC_50_ estimation of chemotherapeutic efficacy in the high‐risk and low‐risk groups in the chemotherapeutic drug MK‐2206. Figure S27: IC_50_ estimation of chemotherapeutic efficacy in the high‐risk and low‐risk groups in the chemotherapeutic drug MK‐8776. Figure S28: IC_50_ estimation of chemotherapeutic efficacy in the high‐risk and low‐risk groups in the chemotherapeutic drug MN‐64. Figure S29: IC_50_ estimation of chemotherapeutic efficacy in the high‐risk and low‐risk groups in the chemotherapeutic drug NU7441. Figure S30: IC_50_ estimation of chemotherapeutic efficacy in the high‐risk and low‐risk groups in the chemotherapeutic drug osimertinib. Figure S31: IC_50_ estimation of chemotherapeutic efficacy in the high‐risk and low‐risk groups in the chemotherapeutic drug paclitaxel. Figure S32: IC_50_ estimation of chemotherapeutic efficacy in the high‐risk and low‐risk groups in the chemotherapeutic drug PD173074. Figure S33: IC_50_ estimation of chemotherapeutic efficacy in the high‐risk and low‐risk groups in the chemotherapeutic drug PD0325901. Figure S34: IC_50_ estimation of chemotherapeutic efficacy in the high‐risk and low‐risk groups in the chemotherapeutic drug acetalax. Figure S35: IC_50_ estimation of chemotherapeutic efficacy in the high‐risk and low‐risk groups in the chemotherapeutic drug afatinib. Figure S36: IC_50_ estimation of chemotherapeutic efficacy in the high‐risk and low‐risk groups in the chemotherapeutic drug afuresertib. Figure S37: IC_50_ estimation of chemotherapeutic efficacy in the high‐risk and low‐risk groups in the chemotherapeutic drug docetaxel. Figure S38: IC_50_ estimation of chemotherapeutic efficacy in the high‐risk and low‐risk groups in the chemotherapeutic drug AGI‐5198. Figure S39: IC_50_ estimation of chemotherapeutic efficacy in the high‐risk and low‐risk groups in the chemotherapeutic drug alpelisib. Figure S40: IC_50_ estimation of chemotherapeutic efficacy in the high‐risk and low‐risk groups in the chemotherapeutic drug axitinib. Figure S41: IC_50_ estimation of chemotherapeutic efficacy in the high‐risk and low‐risk groups in the chemotherapeutic drug AZD5363. Figure S42: IC_50_ estimation of chemotherapeutic efficacy in the high‐risk and low‐risk groups in the chemotherapeutic drug AZD5582. Figure S43: IC_50_ estimation of chemotherapeutic efficacy in the high‐risk and low‐risk groups in the chemotherapeutic drug AZD6738. Figure S44: IC_50_ estimation of chemotherapeutic efficacy in the high‐risk and low‐risk groups in the chemotherapeutic drug AZD7762. Figure S45: IC_50_ estimation of chemotherapeutic efficacy in the high‐risk and low‐risk groups in the chemotherapeutic drug BDP‐00009066. Figure S46: IC_50_ estimation of chemotherapeutic efficacy in the high‐risk and low‐risk groups in the chemotherapeutic drug BMS‐536924. Figure S47: IC_50_ estimation of chemotherapeutic efficacy in the high‐risk and low‐risk groups in the chemotherapeutic drug bortezomib. Figure S48: IC_50_ estimation of chemotherapeutic efficacy in the high‐risk and low‐risk groups in the chemotherapeutic drug BPD‐00008900. Figure S49: IC_50_ estimation of chemotherapeutic efficacy in the high‐risk and low‐risk groups in the chemotherapeutic drug carmustine. Figure S50: IC_50_ estimation of chemotherapeutic efficacy in the high‐risk and low‐risk groups in the chemotherapeutic drug cediranib. Figure S51: IC_50_ estimation of chemotherapeutic efficacy in the high‐risk and low‐risk groups in the chemotherapeutic drug crizotinib. Figure S52: IC_50_ estimation of chemotherapeutic efficacy in the high‐risk and low‐risk groups in the chemotherapeutic drug cyclophosphamide. Figure S53: IC_50_ estimation of chemotherapeutic efficacy in the high‐risk and low‐risk groups in the chemotherapeutic drug CZC24832. Figure S54: IC_50_ estimation of chemotherapeutic efficacy in the high‐risk and low‐risk groups in the chemotherapeutic drug dactinomycin. Figure S55: IC_50_ estimation of chemotherapeutic efficacy in the high‐risk and low‐risk groups in the chemotherapeutic drug daporinad. Figure S56: IC_50_ estimation of chemotherapeutic efficacy in the high‐risk and low‐risk groups in the chemotherapeutic drug dasatinib. Figure S57: IC_50_ estimation of chemotherapeutic efficacy in the high‐risk and low‐risk groups in the chemotherapeutic drug epirubicin. Figure S58: IC_50_ estimation of chemotherapeutic efficacy in the high‐risk and low‐risk groups in the chemotherapeutic drug EPZ004777. Figure S59: IC_50_ estimation of chemotherapeutic efficacy in the high‐risk and low‐risk groups in the chemotherapeutic drug ERK‐6604. Figure S60: IC_50_ estimation of chemotherapeutic efficacy in the high‐risk and low‐risk groups in the chemotherapeutic drug erlotinib. Figure S61: IC_50_ estimation of chemotherapeutic efficacy in the high‐risk and low‐risk groups in the chemotherapeutic drug 5‐fluorouracil. Figure S62: IC_50_ estimation of chemotherapeutic efficacy in the high‐risk and low‐risk groups in the chemotherapeutic drug fulvestrant. Figure S63: IC_50_ estimation of chemotherapeutic efficacy in the high‐risk and low‐risk groups in the chemotherapeutic drug gallibiscoquinazole. Figure S64: IC_50_ estimation of chemotherapeutic efficacy in the high‐risk and low‐risk groups in the chemotherapeutic drug GDC0810. Figure S65: IC_50_ estimation of chemotherapeutic efficacy in the high‐risk and low‐risk groups in the chemotherapeutic drug gefitinib. Figure S66: IC_50_ estimation of chemotherapeutic efficacy in the high‐risk and low‐risk groups in the chemotherapeutic drug GNE‐317. Figure S67: IC_50_ estimation of chemotherapeutic efficacy in the high‐risk and low‐risk groups in the chemotherapeutic drug GSK343. Figure S68: IC_50_ estimation of chemotherapeutic efficacy in the high‐risk and low‐risk groups in the chemotherapeutic drug I‐BRD9. Figure S69: IC_50_ estimation of chemotherapeutic efficacy in the high‐risk and low‐risk groups in the chemotherapeutic drug ipatasertib. Figure S70: IC_50_ estimation of chemotherapeutic efficacy in the high‐risk and low‐risk groups in the chemotherapeutic drug PF‐4708671. Figure S71: IC_50_ estimation of chemotherapeutic efficacy in the high‐risk and low‐risk groups in the chemotherapeutic drug PFI3. Figure S72: IC_50_ estimation of chemotherapeutic efficacy in the high‐risk and low‐risk groups in the chemotherapeutic drug pictilisib. Figure S73: IC_50_ estimation of chemotherapeutic efficacy in the high‐risk and low‐risk groups in the chemotherapeutic drug PLX‐4720. Figure S74: IC_50_ estimation of chemotherapeutic efficacy in the high‐risk and low‐risk groups in the chemotherapeutic drug PRT062607. Figure S75: IC_50_ estimation of chemotherapeutic efficacy in the high‐risk and low‐risk groups in the chemotherapeutic drug ribociclib. Figure S76: IC_50_ estimation of chemotherapeutic efficacy in the high‐risk and low‐risk groups in the chemotherapeutic drug RO‐3306. Figure S77: IC_50_ estimation of chemotherapeutic efficacy in the high‐risk and low‐risk groups in the chemotherapeutic drug RVX‐208. Figure S78: IC_50_ estimation of chemotherapeutic efficacy in the high‐risk and low‐risk groups in the chemotherapeutic drug sapitinib. Figure S79: IC_50_ estimation of chemotherapeutic efficacy in the high‐risk and low‐risk groups in the chemotherapeutic drug SB505124. Figure S80: IC_50_ estimation of chemotherapeutic efficacy in the high‐risk and low‐risk groups in the chemotherapeutic drug SCH772984. Figure S81: IC_50_ estimation of chemotherapeutic efficacy in the high‐risk and low‐risk groups in the chemotherapeutic drug sepantronium. Figure S82: IC_50_ estimation of chemotherapeutic efficacy in the high‐risk and low‐risk groups in the chemotherapeutic drug TAF1‐5496. Figure S83: IC_50_ estimation of chemotherapeutic efficacy in the high‐risk and low‐risk groups in the chemotherapeutic drug talazoparib.

## Data Availability

Relevant datasets are available at https://www.ncbi.nlm.nih.gov/geo under accession number GSE10186.
