# Supplementary material for: Design and Validation of a Predictive Model for Hepatocellular Carcinoma Based on Genes With Differential Expression Driven by DNA Methylation
Source: Int J Genomics. 2026 Jan 27;2026:2729004. doi: 10.1155/ijog/2729004 (PMC12838239; doi:10.1155/ijog/2729004)
Supplement: Supplementary file 1 — Supporting Information Additional supporting information can be found online in the Supporting Information section. Figure S1: IC50 estimation of chemotherapeutic efficacy in the high‐risk and low‐risk groups in the chemotherapeutic drug IWP. Figure S2: IC50 estimation of chemotherapeutic efficacy in the high‐risk and low‐risk groups in the chemotherapeutic drug taselisib. Figure S3: IC50 estimation of chemotherapeutic efficacy in the high‐risk and low‐risk groups in the chemotherapeutic drug telomerase. Figure S4: IC50 estimation of chemotherapeutic efficacy in the high‐risk and low‐risk groups in the chemotherapeutic drug temozolomide. Figure S5: IC50 estimation of chemotherapeutic efficacy in the high‐risk and low‐risk groups in the chemotherapeutic drug trametinib. Figure S6: IC50 estimation of chemotherapeutic efficacy in the high‐risk and low‐risk groups in the chemotherapeutic drug ulixertinib. Figure S7: IC50 estimation of chemotherapeutic efficacy in the high‐risk and low‐risk groups in the chemotherapeutic drug ULK1‐4989. Figure S8: IC50 estimation of chemotherapeutic efficacy in the high‐risk and low‐risk groups in the chemotherapeutic drug UMI‐77. Figure S9: IC50 estimation of chemotherapeutic efficacy in the high‐risk and low‐risk groups in the chemotherapeutic drug VE821. Figure S10: IC50 estimation of chemotherapeutic efficacy in the high‐risk and low‐risk groups in the chemotherapeutic drug VE‐822. Figure S11: IC50 estimation of chemotherapeutic efficacy in the high‐risk and low‐risk groups in the chemotherapeutic drug venetoclax. Figure S12: IC50 estimation of chemotherapeutic efficacy in the high‐risk and low‐risk groups in the chemotherapeutic drug vinblastine. Figure S13: IC50 estimation of chemotherapeutic efficacy in the high‐risk and low‐risk groups in the chemotherapeutic drug vinorelbine. Figure S14: IC50 estimation of chemotherapeutic efficacy in the high‐risk and low‐risk groups in the chemotherapeutic drug VX‐11e. Figure S15: IC50 estimat [file IJOG-2026-2729004-s001.docx]

.
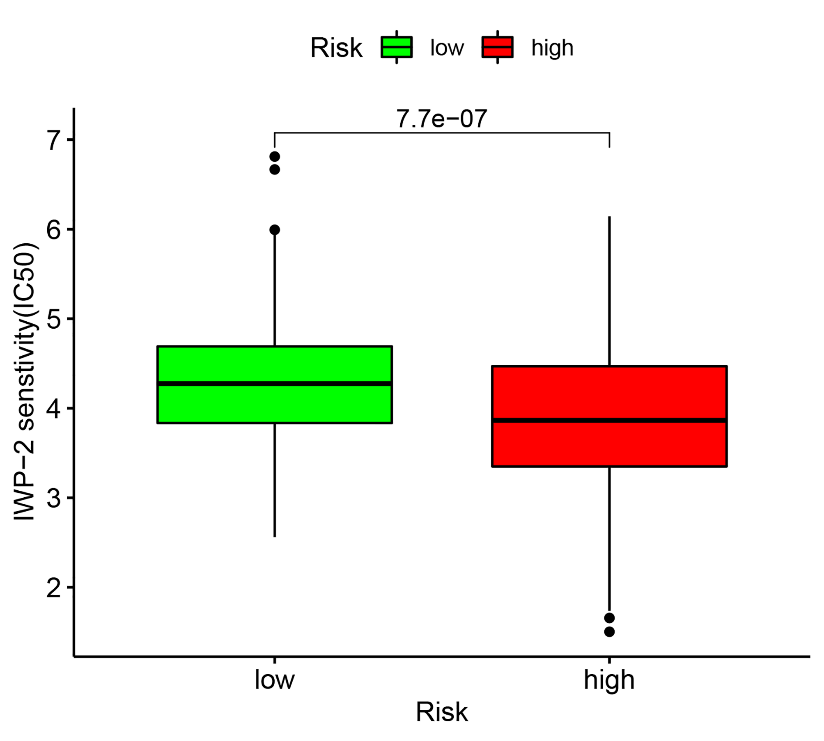


**Figure S1:** IC50 estimation of chemotherapeutic efficacy in high-risk and low-risk groups In the chemotherapeutic drug IWP-2


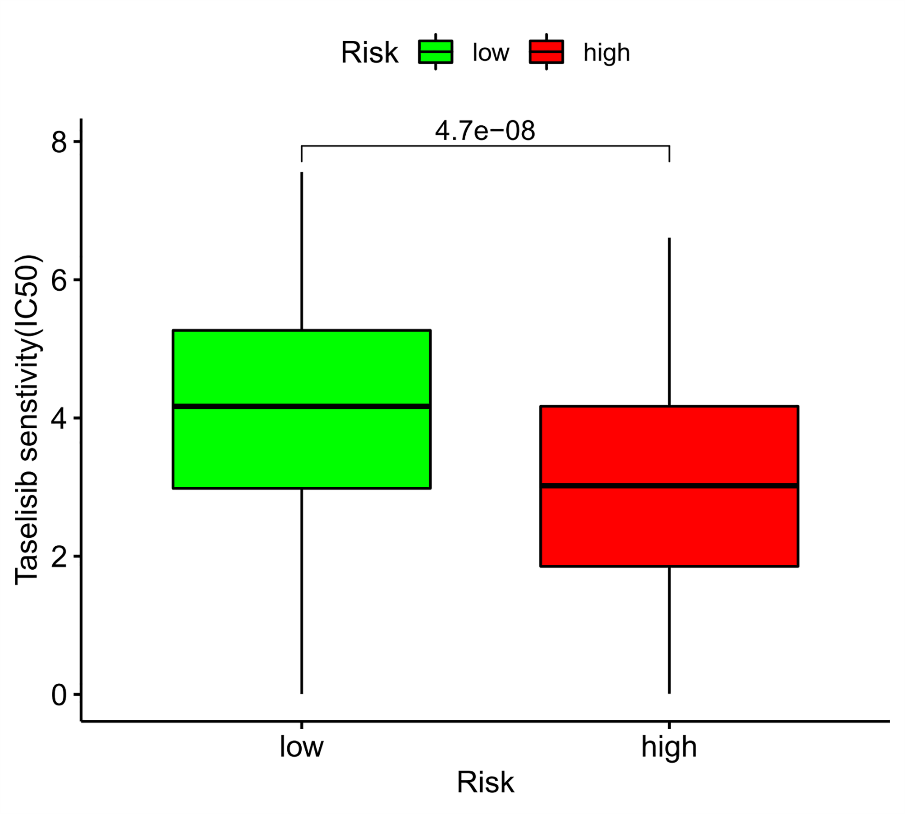


**Figure S2:** IC50 estimation of chemotherapeutic efficacy in high-risk and low-risk groups In the chemotherapeutic drug Taselisib





**Figure S3:** IC50 estimation of chemotherapeutic efficacy in high-risk and low-risk groups In the chemotherapeutic drug Telomerase





**Figure S4:** IC50 estimation of chemotherapeutic efficacy in high-risk and low-risk groups In the chemotherapeutic drug Temozolomide


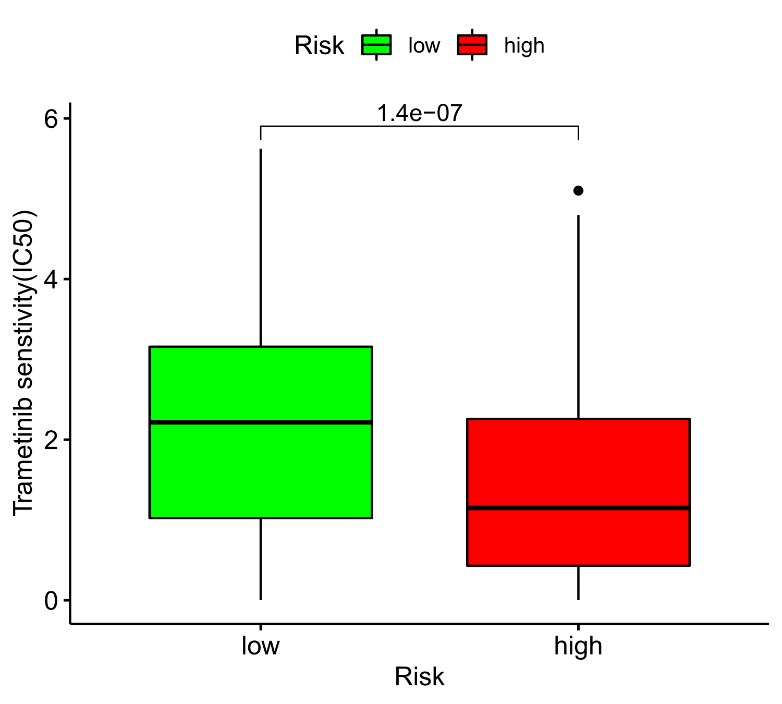


**Figure S5:** IC50 estimation of chemotherapeutic efficacy in high-risk and low-risk groups In the chemotherapeutic drug Trametinib





**Figure S6:** IC50 estimation of chemotherapeutic efficacy in high-risk and low-risk groups In the chemotherapeutic drug Ulixertinib





**Figure S7:** IC50 estimation of chemotherapeutic efficacy in high-risk and low-risk groups In the chemotherapeutic drug ULK1-4989





**Figure S8:** IC50 estimation of chemotherapeutic efficacy in high-risk and low-risk groups In the chemotherapeutic drug UMI-77





**Figure S9:** IC50 estimation of chemotherapeutic efficacy in high-risk and low-risk groups In the chemotherapeutic drug VE821





**Figure S10:** IC50 estimation of chemotherapeutic efficacy in high-risk and low-risk groups In the chemotherapeutic drug VE-822





**Figure S11:** IC50 estimation of chemotherapeutic efficacy in high-risk and low-risk groups In the chemotherapeutic drug Venetoclax





**Figure S12:** IC50 estimation of chemotherapeutic efficacy in high-risk and low-risk groups In the chemotherapeutic drug Vinblastine


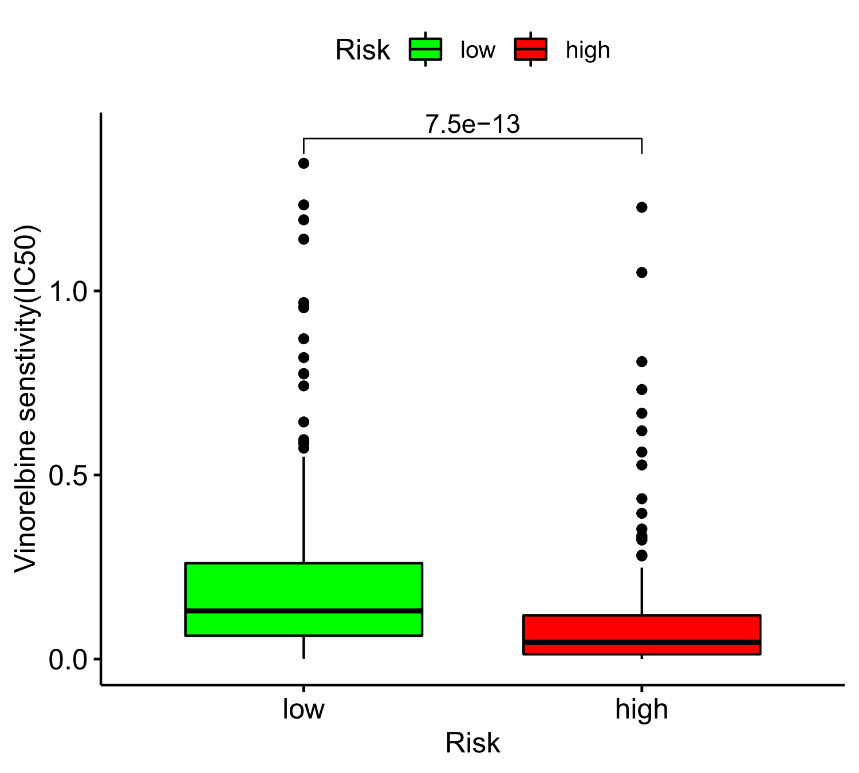


**Figure S13:** IC50 estimation of chemotherapeutic efficacy in high-risk and low-risk groups In the chemotherapeutic drug Vinorelbine





**Figure S14:** IC50 estimation of chemotherapeutic efficacy in high-risk and low-risk groups In the chemotherapeutic drug VX-11e





**Figure S15:** IC50 estimation of chemotherapeutic efficacy in high-risk and low-risk groups In the chemotherapeutic drug Weel lnhibitor





**Figure S16:** IC50 estimation of chemotherapeutic efficacy in high-risk and low-risk groups In the chemotherapeutic drug WEHI-539





**Figure S17:** IC50 estimation of chemotherapeutic efficacy in high-risk and low-risk groups In the chemotherapeutic drug WIKI4





**Figure S18:** IC50 estimation of chemotherapeutic efficacy in high-risk and low-risk groups In the chemotherapeutic drug YK-4-279





**Figure S19:** IC50 estimation of chemotherapeutic efficacy in high-risk and low-risk groups In the chemotherapeutic drug Zoledronate





**Figure S20:** IC50 estimation of chemotherapeutic efficacy in high-risk and low-risk groups In the chemotherapeutic drug JAK1-8709





**Figure S21:** IC50 estimation of chemotherapeutic efficacy in high-risk and low-risk groups In the chemotherapeutic drug Staurosporine





**Figure S22:** IC50 estimation of chemotherapeutic efficacy in high-risk and low-risk groups In the chemotherapeutic drug JQ1





**Figure S23:** IC50 estimation of chemotherapeutic efficacy in high-risk and low-risk groups In the chemotherapeutic drug MIM1





**Figure S24:** IC50 estimation of chemotherapeutic efficacy in high-risk and low-risk groups In the chemotherapeutic drug MIRA-1





**Figure S25:** IC50 estimation of chemotherapeutic efficacy in high-risk and low-risk groups In the chemotherapeutic drug MK-1775





**Figure S26:** IC50 estimation of chemotherapeutic efficacy in high-risk and low-risk groups In the chemotherapeutic drug MK-2206



**Figure S27:** IC50 estimation of chemotherapeutic efficacy in high-risk and low-risk groups In the chemotherapeutic drug MK-8776





**Figure S28:** IC50 estimation of chemotherapeutic efficacy in high-risk and low-risk groups In the chemotherapeutic drug MN-64





**Figure S29:** IC50 estimation of chemotherapeutic efficacy in high-risk and low-risk groups In the chemotherapeutic drug NU7441





**Figure S30:** IC50 estimation of chemotherapeutic efficacy in high-risk and low-risk groups In the chemotherapeutic drug Osimertinib





**Figure S31:** IC50 estimation of chemotherapeutic efficacy in high-risk and low-risk groups In the chemotherapeutic drug Paclitaxel





**Figure S32:** IC50 estimation of chemotherapeutic efficacy in high-risk and low-risk groups In the chemotherapeutic drug PD173074





**Figure S33:** IC50 estimation of chemotherapeutic efficacy in high-risk and low-risk groups In the chemotherapeutic drug PD0325901





**Figure S34:** IC50 estimation of chemotherapeutic efficacy in high-risk and low-risk groups In the chemotherapeutic drug Acetalax





**Figure S35:** IC50 estimation of chemotherapeutic efficacy in high-risk and low-risk groups In the chemotherapeutic drug Afatinib





**Figure S36:** IC50 estimation of chemotherapeutic efficacy in high-risk and low-risk groups In the chemotherapeutic drug Afuresertib





**Figure S37:** IC50 estimation of chemotherapeutic efficacy in high-risk and low-risk groups In the chemotherapeutic drug Docetaxel





**Figure S38:** IC50 estimation of chemotherapeutic efficacy in high-risk and low-risk groups In the chemotherapeutic drug AGI-5198





**Figure S39:** IC50 estimation of chemotherapeutic efficacy in high-risk and low-risk groups In the chemotherapeutic drug Alpelisib





**Figure S40:** IC50 estimation of chemotherapeutic efficacy in high-risk and low-risk groups In the chemotherapeutic drug Axitinib





**Figure S41:** IC50 estimation of chemotherapeutic efficacy in high-risk and low-risk groups In the chemotherapeutic drug AZD5363





**Figure S42:** IC50 estimation of chemotherapeutic efficacy in high-risk and low-risk groups In the chemotherapeutic drug AZD5582





**Figure S43:** IC50 estimation of chemotherapeutic efficacy in high-risk and low-risk groups In the chemotherapeutic drug AZD6738





**Figure S44:** IC50 estimation of chemotherapeutic efficacy in high-risk and low-risk groups In the chemotherapeutic drug AZD7762





**Figure S45:** IC50 estimation of chemotherapeutic efficacy in high-risk and low-risk groups In the chemotherapeutic drug BDP-00009066





**Figure S46:** IC50 estimation of chemotherapeutic efficacy in high-risk and low-risk groups In the chemotherapeutic drug BMS-536924





**Figure S47:** IC50 estimation of chemotherapeutic efficacy in high-risk and low-risk groups In the chemotherapeutic drug Bortezomib





**Figure S48:** IC50 estimation of chemotherapeutic efficacy in high-risk and low-risk groups In the chemotherapeutic drug BPD-00008900





**Figure S49:** IC50 estimation of chemotherapeutic efficacy in high-risk and low-risk groups In the chemotherapeutic drug Carmustine





**Figure S50:** IC50 estimation of chemotherapeutic efficacy in high-risk and low-risk groups In the chemotherapeutic drug Cediranib





**Figure S51:** IC50 estimation of chemotherapeutic efficacy in high-risk and low-risk groups In the chemotherapeutic drug Crizotinib





**Figure S52:** IC50 estimation of chemotherapeutic efficacy in high-risk and low-risk groups In the chemotherapeutic drug Cyclophosphamide





**Figure S53:** IC50 estimation of chemotherapeutic efficacy in high-risk and low-risk groups In the chemotherapeutic drug CZC24832





**Figure S54:** IC50 estimation of chemotherapeutic efficacy in high-risk and low-risk groups In the chemotherapeutic drug Dactinomycin





**Figure S55:** IC50 estimation of chemotherapeutic efficacy in high-risk and low-risk groups In the chemotherapeutic drug Daporinad





**Figure S56:** IC50 estimation of chemotherapeutic efficacy in high-risk and low-risk groups In the chemotherapeutic drug Dasatinib





**Figure S57:** IC50 estimation of chemotherapeutic efficacy in high-risk and low-risk groups In the chemotherapeutic drug Epirubicin





**Figure S58:** IC50 estimation of chemotherapeutic efficacy in high-risk and low-risk groups In the chemotherapeutic drug EPZ004777





**Figure S59:** IC50 estimation of chemotherapeutic efficacy in high-risk and low-risk groups In the chemotherapeutic drug ERK-6604





**Figure S60:** IC50 estimation of chemotherapeutic efficacy in high-risk and low-risk groups In the chemotherapeutic drug Erlotinib





**Figure S61:** IC50 estimation of chemotherapeutic efficacy in high-risk and low-risk groups In the chemotherapeutic drug 5-Fluorouracil





**Figure S62:** IC50 estimation of chemotherapeutic efficacy in high-risk and low-risk groups In the chemotherapeutic drug Fulvestrant





**Figure S63:** IC50 estimation of chemotherapeutic efficacy in high-risk and low-risk groups In the chemotherapeutic drug Gallibiscoquinazole





**Figure S64:** IC50 estimation of chemotherapeutic efficacy in high-risk and low-risk groups In the chemotherapeutic drug GDC0810





**Figure S65:** IC50 estimation of chemotherapeutic efficacy in high-risk and low-risk groups In the chemotherapeutic drug Gefitinib





**Figure S66:** IC50 estimation of chemotherapeutic efficacy in high-risk and low-risk groups In the chemotherapeutic drug GNE-317





**Figure S67:** IC50 estimation of chemotherapeutic efficacy in high-risk and low-risk groups In the chemotherapeutic drug GSK343





**Figure S68:** IC50 estimation of chemotherapeutic efficacy in high-risk and low-risk groups In the chemotherapeutic drug I-BRD9





**Figure S69:** IC50 estimation of chemotherapeutic efficacy in high-risk and low-risk groups In the chemotherapeutic drug Ipatasertib





**Figure S70:** IC50 estimation of chemotherapeutic efficacy in high-risk and low-risk groups In the chemotherapeutic drug PF-4708671





**Figure S71:** IC50 estimation of chemotherapeutic efficacy in high-risk and low-risk groups In the chemotherapeutic drug PFI3





**Figure S72:** IC50 estimation of chemotherapeutic efficacy in high-risk and low-risk groups In the chemotherapeutic drug Pictilisib





**Figure S73:** IC50 estimation of chemotherapeutic efficacy in high-risk and low-risk groups In the chemotherapeutic drug PLX-4720





**Figure S74:** IC50 estimation of chemotherapeutic efficacy in high-risk and low-risk groups In the chemotherapeutic drug PRT062607





**Figure S75:** IC50 estimation of chemotherapeutic efficacy in high-risk and low-risk groups In the chemotherapeutic drug Ribociclib





**Figure S76:** IC50 estimation of chemotherapeutic efficacy in high-risk and low-risk groups In the chemotherapeutic drug RO-3306





**Figure S77:** IC50 estimation of chemotherapeutic efficacy in high-risk and low-risk groups In the chemotherapeutic drug RVX-208





**Figure S78:** IC50 estimation of chemotherapeutic efficacy in high-risk and low-risk groups In the chemotherapeutic drug Sapitinib





**Figure S79:** IC50 estimation of chemotherapeutic efficacy in high-risk and low-risk groups In the chemotherapeutic drug SB505124





**Figure S80:** IC50 estimation of chemotherapeutic efficacy in high-risk and low-risk groups In the chemotherapeutic drug SCH772984





**Figure S81:** IC50 estimation of chemotherapeutic efficacy in high-risk and low-risk groups In the chemotherapeutic drug Sepantronium





**Figure S82:** IC50 estimation of chemotherapeutic efficacy in high-risk and low-risk groups In the chemotherapeutic drug TAF1-5496





**Figure S83:** IC50 estimation of chemotherapeutic efficacy in high-risk and low-risk groups In the chemotherapeutic drug Talazoparib
